# Affinity Sensing Strategies for the Detection of Pesticides in Food

**DOI:** 10.3390/foods7090148

**Published:** 2018-09-05

**Authors:** Denise Capoferri, Flavio Della Pelle, Michele Del Carlo, Dario Compagnone

**Affiliations:** Faculty of Biosciences and Technologies for Food, Agriculture and Environment, University of Teramo, via R. Balzarini 1, 64100 Teramo, Italy; dcapoferri@unite.it (D.C.); fdellapelle@unite.it (F.D.P.); mdelcarlo@unite.it (M.D.C.)

**Keywords:** affinity sensors, pesticides, immunosensors, aptamers, molecularly imprinted polymers, electrochemical detection, optical detection

## Abstract

This is a review of recent affinity-based approaches that detect pesticides in food. The importance of the quantification and monitoring of pesticides is firstly discussed, followed by a description of the different approaches reported in the literature. The different sensing approaches are reported according to the different recognition element used: antibodies, aptamers, or molecularly imprinted polymers. Schemes of detection and the main features of the assays are reported and commented upon. The large number of affinity sensors recently developed and tested on real samples demonstrate that this approach is ready to be validated to monitor the amount of pesticides used in food commodities.

## 1. Introduction

### 1.1. Pesticides

Pesticides are defined, by the Food and Agriculture Organization [1], as any substance or mixture of substances intended for repelling, destroying or controlling any pest causing harm or otherwise interfering during the production, processing, storage, transport or marketing of food, agricultural commodities, animal feeds or products that may be administered to animals for the control of insects, arachnids or other pests in or on their bodies. The term includes substances intended for use as insect or plant growth regulators, defoliants, desiccants, agents for setting, thinning or preventing the premature fall of fruit, and substances applied to crops either before or after harvest to protect the commodities from deterioration during storage and transport [1].

Pesticides can be classified in several ways, according to their toxicity (dangerous, highly dangerous, moderately dangerous, and slightly dangerous) and their median lifetime (permanent, persistent, moderately persistent and not persistent). Often, due to a large number of chemicals and pesticides, they are classified according to the use as insecticides, miticides, herbicides, nematicides, fungicides, molluscicides, and rodenticides [2,3,4]. Referring to the chemical structure, the commonly reported main classes are organochlorines, organophosphates, carbamates, and pyrethroids [5]. In addition to the already commonly reported classes of pesticides, there are other chemical classes employed as triazine herbicides, ureic, hormonal, amides, nitro compounds, benzimidazoles, ftalamidas, bipyridyl compounds, ethylene dibromide, sulfur containing compounds, copper or mercury, among others [2,5]. An idea of the different classes of pesticides, their chemical structure, occurrence in different foods, and the relative European maximum residue limits (MRLs) (in EU) can be found in the EU pesticides database [6]. Moreover, recently, an international database for pesticide risk assessments and management has been reviewed by Lewis et al. [7]. Organochlorine pesticides are probably the most toxic class [8,9], to which some of the oldest compounds employed belong and are characterized by active molecules that generally persist in the environment for more than 30 years. For this reason, over the last years, pesticides such as organochlorines started to be replaced by compounds and formulations characterized by a faster biodegradation, as in the case of organophosphorus pesticides [10]. Organophosphorus and carbamate insecticides are largely used due to their high insecticidal activity and relatively low persistence in the environment [11]. Pesticides are indispensable to the modern agriculture in order to protect crops both in pre and post-harvest phases [12]. Although pesticides are directly applied to plants and soils, only about 1% of pesticide sprayed is delivered to the real target [13]. The intensification of agricultural practices can lead to an accumulation of pesticide residues that pose a serious risk to human health and the environment worldwide [14,15,16], with loss of biodiversity [17] since they are equally toxic or harmful to non-target organisms [5]. Indeed, these compounds are widely distributed in the environment and have been detected in water, soil, sewage sludge, sediments, and the aquatic biota [18,19,20,21,22,23]. Even at low levels, they can cause adverse effects on humans, plants, animals and ecosystems [5]. The European Union, the Codex Alimentarius Commission of the Food and Agriculture Organization of the United Nations (FAO) and the World Health Organization (WHO) have established maximum residue limits (MRLs) for pesticides residues in food [24,25]. MRLs are defined as the maximum concentration of pesticide residue likely to occur in or on food commodities and animal feeds after the use of pesticides according to good agricultural practice (GAP) [26]. Humans can come in contact with pesticides by different routes of exposure like as through the skin, respiratory tract, ingestion [27].

The toxicity of pesticides can be acute or chronic: acute toxicity is due to short-term exposure to relatively large amounts of chemicals and is revealed within a relatively short period of time; chronic toxicity is due to long-term and low-level exposure to chemicals and can be revealed over a longer period of time. Systematic review and meta-analyses, have recently confirmed that exposure to pesticides can lead to numerous diseases [28]. In human beings, they may increase the risk of psychiatric [29], endocrine-disrupting disorders [30] and promote renal, neurological, hepatic and reproductive problems, even at low levels [31,32,33]. The implications of pesticide residues for human health and the environment have been reviewed recently in different studies [34,35]. However, pesticides will continue to play an important role in plant diseases prevention and pest management. In developing countries, where farmers are shifting from subsistence agriculture to modern agriculture, the use of pesticides may increase at a high rate, and the trend in the use is expected to increase substantially in the next few decades [25,36,37,38]. Therefore, their use should be optimized and rationalized, considering the safety of producers and consumers as well as the environmental impact [39].

### 1.2. Pesticides Detection and Affinity Sensors

The monitoring and the rapid quantification of pesticides and their residues have become extremely important to ensure compliance with legal limits [13,40,41]. Moreover, the analysis of these compounds is an important issue for their potential bioaccumulation, high toxicity and their long-term damage risk, also for the use at low concentration [27]. Food quality and safety assurance require fast and easy analytical tools [42,43,44,45] to work alongside confirmatory methods such as chromatography coupled to mass spectrometry [46,47]. The main features of sensors are: selectivity, that allows direct detection of the analyte without laborious sample pretreatment or with minimal sample pretreatment [44,47,48]; fast analysis, with results in few minutes; low costs; potential of miniaturization and portability, which makes in situ and real time monitoring possible. Furthermore, sensors are, generally, easy to use and do not require highly trained personnel [42].

Affinity sensors are based on the selective recognition of the analyte via a biological/biomimetic element such as antigen–antibody binding, nucleic acid hybridization or synthetic receptor/target recognition (e.g., molecular imprinted polymers (MIPs), aptamers) [49]. On the basis of the recognition element, the affinity sensors can be classified as immunosensors, DNA sensors, aptasensors, MIPs sensors [49]. Affinity sensing is mostly carried out using two assay formats, competitive or non-competitive; usually the choice is made on the basis of the molecular size of the analyte. In particular, for analytes holding at least two binding sites (epitopes when an antibody is used) the non-competitive approach is chosen whereas for smaller analytes a competitive approach is generally preferred.

Affinity sensors can be label free or labeled with enzymes, nanoparticles, fluorescent or electroluminescent probes. Affinity strategies and their applications were reviewed in the last years by several authors [49,50,51].

The aim of this review is to give a general overview on the most recent development of affinity sensors for the detection of pesticides in food. In particular, the discussion will be focused on electrochemical (i.e., voltammetric, amperometric, potentiometric, and impedimetric) and optical (colorimetric, fluorescent, Surface Plasmon Resonance based, Surface-enhanced Raman Scattering based) immunosensors, aptasensors, and MIPs sensors.

## 2. Immunosensors

Antibodies (Ab) are a class of proteins that respond to the presence of foreign substances called antigens (Ag). In nature antibodies are produced in response to antigens, such as toxins, chemicals, drugs, virus particles, bacterial toxins and other foreign substances [50]. Immunosensors are known as affinity ligand-based biosensors that record immunochemical interactions between immobilized antibodies (Ab)/antigens (Ag) on the transducer surface and can provide concentration-dependent signals [12]. According to the transduction strategies used, immunosensors can be classified in electrochemical [52,53,54], optical and piezoelectrical sensors [53]. The use of the immunosensors for pesticides detection in food and environmental samples has been already discussed in previous reviews [54,55,56,57]. Recent advancement in the immunosensors field are here reported according to the transduction strategies employed.

**Figure 1:**
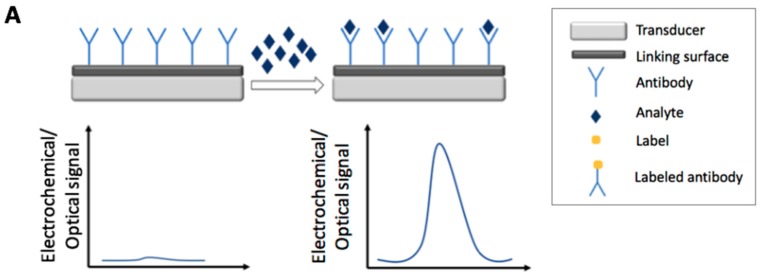

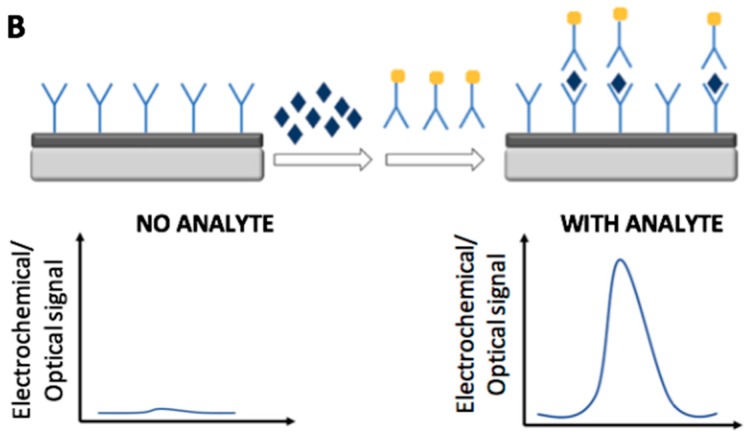
Schematic representation of electrochemical and optical non-competitive detection strategies by the use of immunosensors: label free assay (**A**) and labeled assay (**B**).

**Figure 2:**
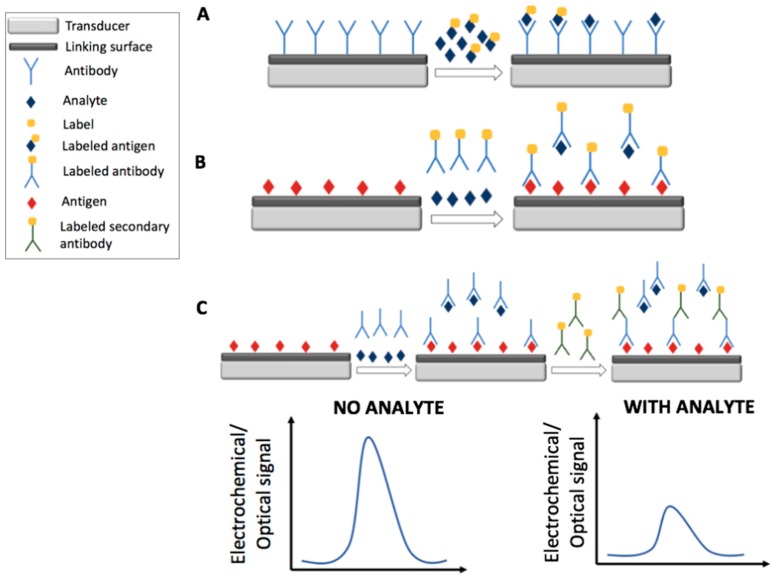
Schematic representation of electrochemical and optical labeled competitive detection strategies by the use of immunosensors: competition for the antibody among the analyte and the labeled antigen (**A**); competition for the labeled antibody among the analyte and the antigen (**B**); competition for the antibody among the analyte and the antigen and detection by the use of a labeled secondary antibody (**C**).

Figure 1 and Figure 2 report the main electrochemical and optical detection strategies used for the recognition/quantification of the analytes by the use of immunosensors: in particular label free non-competitive assay (Figure 1A), labeled non-competitive assay (Figure 1B) and labeled competitive assay (Figure 2).

### 2.1. Electrochemical Immunosensors

During the last five years, different sensing strategies for the electrochemical detection of pesticides in food using immunosensors have been developed. Table 1 summarizes the strategies and features of the electrochemical immunosensors discussed in this section. The most used approach for the label-free and non-competitive pesticides detection was Electrochemical Impedance Spectroscopy (EIS) [58,59,60,61,62,63]. In EIS the detection of the analyte is carried out indirectly through the impedimetric response of an electrochemical probe with known and stable electrochemical behavior (e.g., ferrocyanide/ferricyanide redox couple). The impedance response of the sensor increases with the increase of the pesticide concentration due to the hindering effect of Ab-Ag complex towards the redox probe. This ‘diffusion limitation’ leads to an increase of the resistance (analytical signal) of the electron transfer at the sensor surface. Other electrochemical techniques widely employed have been Differential Pulse Voltammetry (DPV) [64], Cyclic Voltammetry (CV) [65,66,67,68,69], Square Wave Voltammetry (SWV) [70,71,72], and amperometry [73]. In voltammetric analysis the information about the analyte is obtained by measuring the current as the potential is varied, while amperometric techniques involve measuring electric currents at fixed potential. For electroactive analytes, the current response increases with concentration and the quantification is directly achieved; non-electroactive analytes (or non-electroactive at the experimental working conditions and potentials used) are indirectly quantified via redox probes. In the following paragraphs, recent electrochemical immunosensors, classified according to the transduction technique, are reported.

#### 2.1.1. Impedimetric Immunosensors

Electrochemical Impedance Spectroscopy (EIS) is a technique used for the electrochemical characterization of surfaces. The electrical response of the system following the application of a periodic small amplitude alternating current (ac) signal in a wide range of frequencies is recorded. This technique allows to detect small changes on the electroactive surface studied caused by affinity interactions.

Wang et al. [58] prepared a simple impedimetric immunosensor for the selective detection of fenvalerate by modifying a glassy carbon electrode (GCE) with chitosan and glutaraldehyde to immobilize antibodies. The detection limit achieved was 1.91 × 10^−9^ M. The immunosensor was applied in tea samples with an excellent recovery of 103%.

Liu et al. [59] developed an impedimetric immunosensor for the sensitive detection of carbofuran by immobilizing Abs on L-cysteine modified gold electrodes. The limit of detection (LOD) obtained was 4.52 × 10^−10^ M. This method was successfully applied to detect carbofuran in spiked tomato, cabbage, and lettuce samples with an excellent recovery range 90–106%, demonstrating that the sensor can be applied to the detection of carbofuran in real samples.

Impedimetric immunosensors obtained on gold electrode surfaces and based on an interdigitated array of microelectrodes (IDAMs) for the detection of chlorpyrifos were also described. Accordingly, a sensor based on microfluidic chip was developed by Jia et al. [60]. The microelectrode surface was modified with polydiallyldimethylammonium chloride (PDDA) and gold nanoparticles (AuNPs) and the antibody immobilized onto the IDAM surface via protein A. The use of a microfluidic chip with embedded IDAM reduced the amount of reagents, particularly expensive antibodies, and improved the sensitivity. The detection limit was estimated to be 1.43 × 10^−9^ M. In order to test the feasibility of the sensor, spiked vegetable samples were analyzed and recoveries varying in the 75.2% to 96.5% range were achieved. The impedimetric IDAM immunosensor developed by Cao et al. [61] did not use AuNPs and was found more sensitive with a lower detection limit (3.99 × 10^−11^ M) compared to the IDAM immunosensor described above. The proposed immunosensor was applied for the detection of chlorpyrifos in real vegetable samples and similar recovery range, 87.6–96.5%, was obtained.

The use of screen-printed electrodes (SPEs) modified with nanomaterials and nanocomposites for the development of impedimetric immunosensors has been also reported. Fusco et al. [62] presented an immunosensor for the detection of the 2,4-dichlorophenoxy acetic acid (2,4-D) herbicide. The nanocomposite gold nanoparticles-poly-(aniline-*co*-3-aminobenzoic acid)-multi-walled carbon nanotubes (AuNPs-PANABA-MWCNTs) was synthesized by electrochemical polymerization of aniline (ANI) and 3-aminobenzoic acid (3-ABA), in the presence of a dispersion of AuNPs, onto a MWCNTs-based screen-printed electrode. The immunosensor had a LOD of 1.36 × 10^−9^ M; this method was successfully applied to detect 2,4-D in spiked tap water samples with good recovery from 82.0% to 120%. A graphene based immunosensor for highly sensitive detection of parathion was fabricated by Mehta et al. [63]. Screen-printed carbon electrodes were modified with graphene sheets and were then functionalized with 2-aminobenzyl amine (2-ABA) via an electrochemical reaction. The amine functionalized graphene electrodes were then conjugated with the anti-parathion antibodies. The sensor detected parathion in a broad linear concentration range, (3.43 × 10^−13^–3.43 × 10^−9^) M, with a very low limit of detection (1.79 × 10^−13^ M) compared to the work above reported. The usability of the sensor was demonstrated by detecting parathion in spiked tomato and carrot samples. Moreover, the sensor stored at 4 °C gave a response that was fairly stable even after 50 days of storage, showing good stability.

#### 2.1.2. Voltammetric and Amperometric Immunosensors

CV, DPV, and SWV are amongst the most used electroanalytical techniques; in CV a potential is applied to the working electrode at a constant scan rate in the forward and reverse directions, while in DPV the potential is varied using pulses of increasing amplitude and the current is sampled in differential mode. DPV is inherently more sensitive than CV since the contribution to the signal of the capacitive background current is strongly reduced. SWV implies a sequence of potential steps that include the alternant application of oxidation-reduction potential over an increasing ramp of potential; this technique is particularly useful when a reversible electrochemical species is measured, leading to higher sensitivity with respect to DPV.

A simple sensor for atrazine detection via a redox probe (ferrocyanide/ferricyanide redox couple) using DPV was developed by Liu et al. [64] by immobilization of AuNPs onto a gold electrode surface. AuNPs have a larger specific surface area, desirable biocompatibility, and a high surface free energy. With these features, the AuNPs are able to adsorb more antibodies without loss of their biological activity. The peak current of the redox probe decreased with the increase of the atrazine concentration. This was attributed to the formation of the immunocomplex that blocks the electron transfer. A good limit of detection of 7.42 × 10^−11^ M and high recoveries, between 95.5–120%, in maize samples were obtained.

Recently, several authors employed nanocomposites for the production of label-free and non-competitive electrochemical immunosensors. Nanocomposites enhance conductivity and increase the specific surface area of the immunosensor interface resulting in improved immobilization of antibodies. Sun et al. [65] developed an immunosensor for the CV determination of chlorpyrifos by immobilizing an antibody onto a GCE modified with a polyaniline/carboxylated MWCNTs-chitosan nanocomposite (PANI/MWCNTs-CS/GCE) using AuNPs as linker. The immunosensor exhibited a broad linear range from 2.85 × 10^−10^ M to 1.43 × 10^−6^ M, with a detection limit of 1.71 × 10^−10^ M. In order to test the applicability of the sensor, spiked vegetable samples were analyzed and a recovery between 80.6–109% was obtained. Qiao et al. [66] exploited an immunosensor based on a graphene sheets-methylene blue nanocomposite and AuNPs (GS-MB/AuNPs). The proposed sensor had a good detection limit of 1.6 × 10^−10^ M and was used to detect chlorpyrifos by CV in vegetable samples with recovery ranging from 86.0% to 105%. Zhu et al. [67] developed an immunosensor for the rapid detection of carbofuran by CV using MWCNTs and a graphene sheets-ethyleneimine polymer-Au (GS-PEI-Au) nanocomposite built onto the surface of a GCE via self-assembly. The monoclonal antibody against carbofuran was covalently immobilized onto AuNPs, then, the modified electrode was coated with gold nanoparticles-antibody conjugate (AuNPs-Ab). The immunosensor had a detection limit of 1.36 × 10^−10^ M. Spiked vegetable samples were analyzed and the recovery range obtained was 86.0–103%.

The use of enzymes or nanoparticles to label antibodies for labeled and competitive detection of pesticides has been also widely reported. Giannetto et al. [68] realized a competitive immunosensor for atrazine, based on the immobilization of a conjugate atrazine-bovine serum albumine (ATR-BSA) onto a nanostructured gold substrate previously functionalized with poliamidoaminic dendrimers (PAMAM). ATR-BSA conjugate was immobilized on the electrode for the development of a competitive assay. In order to detect the Ab retained onto the electrode surface the immunosensor was incubated with a secondary horseradish peroxidase-labeled antibody (Ab-HRP). The CV response of the developed immunosensor for atrazine was explored over the (4.64 × 10^−11^–4.64 × 10^−6^) M range and a limit of detection of 5.56 × 10^−9^ M was achieved. Spiked corn flakes samples were analyzed and the recovery was in the 109–114% range. Moreover, no significant performance loss was noticed during six-months, with respect to freshly prepared devices. A nanoparticles modified immunosensor for chlorpyrifos detection by CV was realized by Wang et al. [69]. An Indium Tin Oxide (ITO) working electrode was coated with Co_3_O_4_/Polyaniline (Co_3_O_4_/PANI) nanoparticles thin layer. The competitive immunoassay was carried out using chlorpyrifos-BSA (artificial antigen) coupled to the surface of ITO working electrode. The sensor had a broad linear range, (0–2.85 × 10^−5^) M, with a limit of detection of 2.85 × 10^−8^ M. Recoveries obtained with spiked green vegetables and apples were between 82.8–107%.

Liu and coworkers [70,71] reported immunosensors for label-free and non-competitive detection of pesticides by the use of ferrocenedimethylamine (FDMA) and pyrroloquinoline quinone (PQQ) as redox probes. The formation of the complex between surface-bound epitope and antibody blocked counterions accessing the redox probes with a corresponding decrease in SWV current. Because of the affinity between the antibody on the sensing interface and the analyte in solution, the antibody can dissociate from epitope resulting in the increase of SWV current from redox probe. Single wall nanotubes (SWNTs) modified GCE for the detection of paraoxon [71] was obtained derivatizing SWNTs with the redox probe ferrocenedimethylamine (FDMA), the epitope (paraoxon hapten) and the antibody. A detection limit of 7.27 × 10^−9^ M was obtained. Spiked tap and purified water samples were analyzed and good recoveries were obtained (92.0% for tap water and 95.0% for purified water). The other immunosensor reported [70] is a multianalyte sensor for the simultaneous selective detection of endosulfan and paraoxon based on the assembly of SWNTs formed using micro contact printing (MCP) on glassy carbon substrates. The redox probes ferrocenedimethylamine (FDMA) and pyrroloquinoline quinone (PQQ) were attached to the SWNTs modified interface, respectively followed by the attachment of the corresponding epitopes and antibodies. The limits of detection obtained were 1.23 × 10^−10^ M for endosulfan and 7.27 × 10^−9^ M for paraoxon. Spiked tap and purified water samples were analyzed and satisfactory recovery ranges were obtained, in particular 95.0–96.0% and 97.0–98.0% for the tap water and purified water, respectively.

Valera et al. [72] developed an immunosensor based on semiconductor nanocrystals to detect paraquat residues. The immunosensor used electrochemical nanoprobes prepared by labelling the antibodies with CdS nanoparticles (*CdS*NPs) and antigen biofunctionalized magnetic μ-particles. SWV measurements were performed using graphite composite electrodes (GECs). After the immunochemical reaction, the *CdS*NPs were dissolved and the metal ions released reduced at the electrode surface. The signal recorded was in this case inversely proportional to the concentration of pesticide. The LOD obtained was of 3.11 × 10^−11^ M. Combined with a simple extraction procedure of the potato samples assayed, paraquat could be detected (LOD = 5.44 × 10^−9^ M) one order of magnitude below the MRL (set by the EU in this food commodity) with a recovery range of 76.0–97.0%.

Another immunosensor for the detection of paraquat in potato samples, has been reported by Garcia-Febrero et al. [73] using a magnetic graphite-epoxy composite (m-GEC) electrode and antigen biofunctionalized magnetic micro-particles (PQ1-BSAMP). The amperometric signal was provided by an enzymatic probe prepared by covalently linking an enzyme to the specific antibodies (Ab198-cc-HRP). The immunocomplexes formed on top of the modified magnetic micro-particles are captured by the m-GEC, which acts simultaneously as transducer. Paraquat was detected at a concentration of 7.00 × 10^−10^ M. Combined with an effective extraction procedure, paraquat residues were directly detected and accurately quantified in potato samples, with recoveries from 91.8% to 144%. The immunosensor used antigen biofunctionalized magnetic beads (PQ1-BSAMP) and a magnetic electrode to extract the immunocomplexes from the extract, minimizing the potential nonspecific interferences caused by the matrix on the amperometric signal and allowing the direct use on potato extracts without any additional clean-up or purification steps.

### 2.2. Optical Immunosensors

Optical immunosensors for the detection of pesticides in food have been widely employed. In the following paragraphs, recent optical immunosensors, classified according to the transduction technique, are reported. Among the discussed transductions, the most commonly employed optical technique was Surface Plasmon Resonance (SPR) followed by fluorescence and colorimetry. In Table 2 the strategies and features of optical immunosensors discussed in this section have been summarized.

#### 2.2.1. Surface Plasmon Resonance-Based Immunosensors

SPR occurs when polarized light illuminates, under conditions of total reflection, a thin conducting film at the interface between two transparent media with different refractive indexes. It involves the capture of photons by free electrons in the film, and results in reduced reflection at a specific angle, called the SPR angle (θ), which depends on the refractive index near the biosensor surface that is altered by affinity-pair interaction. Indeed, SPR immunosensors measure the mass concentration changes (as a refractive index change) [80] caused by binding of an analyte (or antibody) to the corresponding antibody (or antigen) immobilized onto the sensor surface [81]. The change in mass concentration result in a shift in the SPR angle, that results to be the sensor signal. Tomassetti et al. [74] developed a SPR immunodevice for triazine determination; the proposed device was compared with two amperometric immunosensors. The LOD value was found to be 5.30 × 10^−8^ M and recovery tests were carried out on bovine milk samples, obtaining satisfactory recovery ranges: 94.9–103% for the atrazine and 92.6–95.8% for other triazinic pesticides. However, the developed SPR-based method did not seem to offer substantial advantages compared to the amperometric immunosensors. Nevertheless, the measurement resulted faster and easier to perform.

Guo et al. [75] described a non-competitive immunoassay for trace detection of triazophos using a direct SPR biosensor. The anti-triazophos monoclonal antibodies (mAb) were covalently immobilized onto a sensor chip, coated with a high density carboxymethylated dextran, via amine coupling chemistry. The response (RU resonance unit) increased with the increase of the pesticide concentration. The biosensor assay had a detection limit of 3.06 × 10^−10^ M. The sensor can be re-used for 160 cycles. Moreover, the device was employed to determine triazophos, coupled to the classical QuEChERS (quick, easy, cheap, effective, rugged, and safe) extraction, in spiked vegetable samples. Recoveries from 84.4% to 109% were obtained. Two immunosensors based on the SPR detection of fungicides have been presented by Hirakawa and co-workers [76,77]. In particular, an immunosensor for the detection of the fungicide boscalid in horticultural crops was developed and compared with a direct competitive enzyme-linked immunosorbent assay (dc-ELISA) [76]. The sensor chip was coated with carboxymethyl dextran; then the hapten-bovine serum albumin (hapten-BSA) solution was flowed through the system to bind the chip’s activated carboxyl groups. The pesticide-Ab complex solution was flushed through the sensor system to allow the free Ab binding with the hapten-BSA immobilized on the sensor surface. Recovery of boscalid in spiked horticultural crops was 85.0–109%, indicating the applicability of the sensor. Furthermore, the SPR-sensor exhibited performance comparable with the dc-ELISA, except for the sensitivity. Another immunosensor based on SPR was developed by the same group [77] to analyze chlorothalonil fungicides residues. In this case the SPR-sensor could determine chlorothalonil residues in vegetables at concentrations around the MRLs.

#### 2.2.2. Fluorescence-Based and Colorimetric Immunosensors

Fluorescence is an emission phenomenon in which a fluorophore absorbs light (or electromagnetic radiation) and emits light into the visible spectrum with different (compared with the excitation) wavelength [80]. Zhang et al. [78] designed a simple and highly sensitive immunoassay based on a competitive binding and bio-barcode amplification for the detection of triazophos. Gold nanoparticles were functionalized with antibodies and 6-carboxyfluorescein labeled single-stranded thiol-oligonucleotides (6-FAM-SH-ssDNAs); the fluorescence of 6-FAM was quenched by AuNPs. The fluorescence intensity results inversely proportional to analyte concentration. The linear range of the method obtained was (3.19 × 10^−11^–6.38 × 10^−8^) M with a LOD of 1.92 × 10^−11^ M. The competitive fluorescence bio-barcode immunoassay was applied to tap water, rice, cucumber, cabbage, and apple samples. The pretreatments of solid samples were carried out according to the QuEChERS method with slight modifications; the recovery obtained ranged from 85.0% to 110%.

In colorimetric methods the detection is carried out through analyte-induced color changes. Visual assessment or a simple portable spectrometer make possible a potential on-site simple detection. A flexible, simple and cost-effective nitrocellulose membrane-based colorimetric immunochip assay for the simultaneous detection of seven pesticides from six different chemical groups (triazophos, methyl-parathion, fenpropathrin, carbofuran, thiacloprid, chlorothalonil, and carbendazim) was developed by Lan et al. [79]. The method is based on a secondary Ab-gold conjugate as universal reporter and AuNPs deposition for signal enhancement. The immunochip test was based on the competitive inhibitory interaction. They compared two competitive models: direct model (primary Ab-AuNP conjugate) and indirect model (secondary Ab-AuNP conjugate). An integrated 7-plex immunochip assay based on the indirect model was finally proposed. The detection limits obtained for the studied pesticides ranged from 6.38 × 10^−11^ M to 2.55 × 10^−8^ M. Qualitative results were obtained visually, while semi-quantification of target analytes was easily achieved by grayscale image acquisition using a desktop scanner. In order to assess the usability of the immunochip assay, a multi-residue analysis of seven pesticides was carried out for spiked vegetables and fruits; recoveries ranging from 73.9% to 116% were obtained. Also, in this case, the QuEChERS method was suggested for food sample pretreatment.

## 3. Aptasensors

The etymology of the word ‘aptamer’ come from the Latin word “aptus” meaning “to fit” [82]. The aptamers are short and single-stranded DNA or RNA sequences able to selectively bind low molecular weight organic (or inorganic) substrates or relatively big macro-molecules [83]. Aptamers exhibit binding affinities and specificities comparable in some cases to monoclonal antibodies [84]. Classically, aptamers are selected in vitro using a technique called SELEX (selection evolution of ligands by exponential enrichment) [82,85], starting from synthetic oligonucleotide libraries. Aptamers have been selected to bind/interact with a wide range of molecules as drugs, mycotoxins, pesticides, proteins, up to bacteria [86,87]. They are able to undergo changes in sequence, three-dimensional structure and folding pattern upon binding the target; these properties allow them to better embrace and retain the molecules. Aptamers possess several competitive advantages over antibodies, including: accurate and reproducible chemical production [88], wider detection ranges, higher stability under different chemical and physical conditions, longer shelf life, easy and cost-effective synthesis processes [89], simplicity of modification to obtain various labelled probe elements [90]. Recently, different authors have reviewed the application of aptasensors for detection of different pollutants and contaminants, such as pesticides, in food and environment [83,86,87,91,92,93,94,95]. Recent advancement in the aptasensors field are here reported, classified according to the transduction strategies employed.

### 3.1. Electrochemical Aptasensors

**Figure 3:**
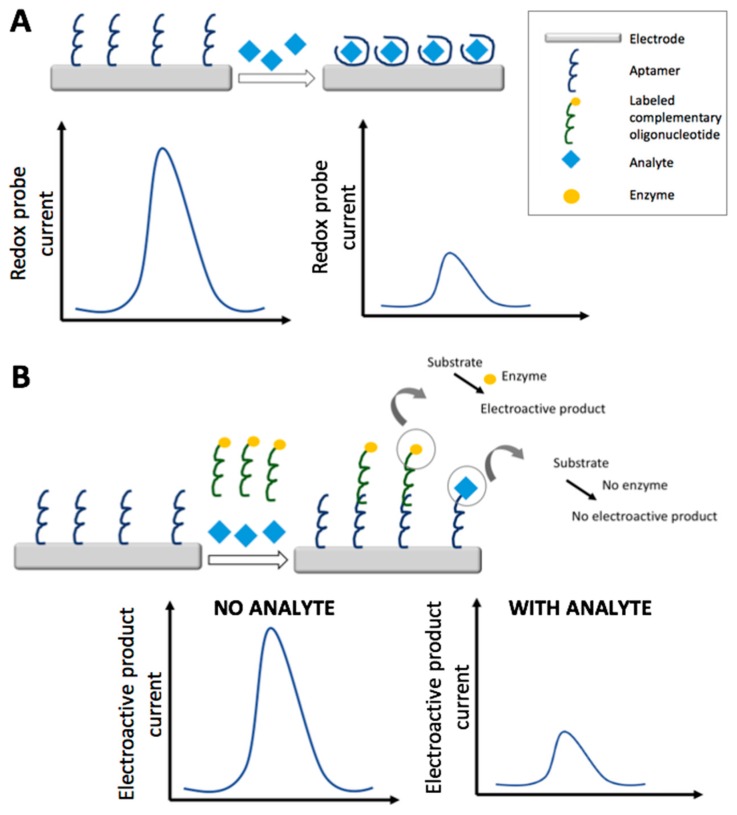
Schematic representation of detection strategies for an electrochemical aptasensor: label free non-competitive detection by the use of a redox probe (**A**); labeled competitive detection exploiting the signal generated by the electroactive product of the reaction catalyzed by an enzyme (**B**). The competition for the aptamer is among the analyte and the labeled complementary oligonucleotide; with the increase of the analyte concentration there is a decrease of the labeled complementary oligonucleotide bound to the aptamer and thus of enzyme and consequently a decrease in the electroactive product signal.

In the following paragraphs, recent electrochemical aptasensors based on the impedimetric and voltammetric transduction techniques are discussed; strategies and features are summarized in Table 3. Figure 3 schematizes the electrochemical detection strategies of a generic electrochemical aptasensor: in particular, label free non-competitive detection (A) and labeled competitive detection (B).

#### 3.1.1. Impedimetric Aptasensors

Electrochemical Impedance Spectroscopy (EIS) is classically employed for electrochemical aptasensors as for the previously reported immunosensors. The formation of the aptamer-pesticide complex produces a conformational change of the aptamers that alters (reduces) the access of an electrochemical probe onto the aptasensor surface. The ‘diffusion limitation’ (due to the aptamer-pesticide complex) leads to an increase of the resistance at the sensor surface, this resistance increase is used as an analytical signal.

Eissa and Zourob [96] developed a simple impedimetric aptasensor, in which a thiol-modified aptamer was immobilized on a gold electrode, for the rapid and low-cost detection of carbendazim with a limit of detection of 4.29 × 10^−11^ M. The proposed aptasensor has been applied in different spiked food matrices (soya milk, mango juice, tomato, and plum fruit). Another impedimetric aptasensor obtained onto a gold electrode was developed by Fan [97], in this case, the thiol-terminated aptamer was immobilized onto AuNPs previously electrodeposited on the bare gold electrode surface by CV. The developed aptasensor exhibited a detection limit of 1.00 × 10^−9^ M. The applicability of the developed aptasensor has been successfully evaluated by determining acetamiprid in tomatoes and the recovery range obtained was between 85.8–105%. Madianos et al. [98] detected acetamiprid and atrazine using aptamer-modified platinum nanoparticles based microwires (PtNPs microwires). By employing sputtering and e-beam lithography, PtNPs were deposited in a bridge-like arrangement, onto interdigitated electrodes (IDEs). The resulting PtNPs microwires were chemically functionalized with (3-glycidyloxypropyl)triethoxysilane (GOPTS). The biosensing platform facilitated charge transfer through the microwire-bridged IDEs, while upon analyte binding to the immobilized aptamers electron transfer was hindered, resulting in an increase of the electrochemical cell impedance. The combination of PtNPs microwires and aptamers allowed the detection of acetamiprid and atrazine with wide linear ranges of (1.00 × 10^−11^–1.00 × 10^−7^) M and (1.00 × 10^−10^–1.00 × 10^−6^) M, respectively. Analytical performance was tested in tap and bottled mineral water; recoveries were between 79.0% and 113%. With the purpose of enhancing the sensitivity of aptasensors for the detection of acetamiprid, some authors proposed the use of nanocomposites that provide a large accessible surface area for loading a large amount of aptamers. This, in theory, should amplify the response signals. Accordingly, an impedimetric aptasensor based on AgNPs decorated nitrogen doped graphene (NG) nanocomposite has been proposed by Jiang et al. [99]. The presented aptasensor exhibited a broad linear range from 1.00 × 10^−13^ M to 5.00 × 10^−9^ M with an extremely low detection limit of 3.30 × 10^−14^ M. Spiked cucumber and tomato samples were analyzed with a recovery range of 86.4–109%.

#### 3.1.2. Voltammetric Aptasensors

Voltammetric detection of electroactive pesticides, by aptasensors, can be directly employed for the electrochemical detection, since current is expected to increase upon binding. Instead, for non-electroactive pesticides (or non-electroactive at the experimental working conditions and potentials used), the indirect quantification can be carried out by the use of an external redox probe. In this case, the current response of the redox probe decreases with the increase of the pesticide concentration, because of the aptamer-pesticide complex formation. Another possible detection strategy is the use of an enzyme as label, exploiting the response of the electroactive enzymatic product, as reported by Rapini et al. [100]. They proposed an aptasensor for acetamiprid, based on competitive labeled format on screen-printed electrode arrays. A streptavidin-alkaline phosphatase conjugate was used as tracer and the enzymatic product was detected by DPV. The limit of detection obtained was 8.60 × 10^−8^ M. The aptasensor was successfully applied for the detection of acetamiprid in spiked fruit juice samples, with a simple pretreatment (filtration and dilution), showing a recovery range between 72.5–110%.

Prabhakar et al. [101] developed a label free non-competitive aptasensor, based onto a chitosan-iron oxide nanocomposite (CHIT-IO) film deposited on fluorine tin oxide (FTO) for the detection of malathion by DPV using a redox probe (ferrocyanide/ferricyanide redox couple). The sensor exhibited linear response for malathion in a wide concentration range, from 3.03 × 10^−12^ M to 3.03 × 10^−8^ M, with a low LOD (3.03 × 10^−12^ M). A recovery range from 80.0% to 88.0% was obtained for lettuce leaves spiked samples.

Xu et al. [102] developed an aptasensor for chlorpyrifos detection based on copper oxide nanoflowers and single-walled carbon nanotubes (CuO NFs-SWCNTs) onto a GCE. The DPV of methylene blue (MB), a redox indicator for DNA hybridization, was applied to monitor the reaction. After the addition of chlorpyrifos, the formation of more compact aptamer-chlorpyrifos complex structure resulted in the decrease of MB bound to guanine bases in the aptamer domain. Consequently, aptamer was forced to dissociate from semi-duplex, resulting in the fleeing of MB from the sensing interface. Thus, the DPV peak current of MB decreased with the increase of chlorpyrifos concentration. The sensor exhibited a detection limit of 2.00 × 10^−10^ M. This aptasensor was successfully applied for the determination of chlorpyrifos in spiked apple and celery cabbage with excellent recoveries from 96.0% to 107%. In addition, the sensor could be regenerated by urea.

Two label-free non-competitive aptasensors for the detection of chlorpyrifos by CV using a redox probe (ferrocyanide/ferricyanide redox couple) were reported by Jiao and co-workers [103,104]. In the first work [103], in order to improve the sensitivity of the aptasensor, the GCE surface was modified with mesoporous carbon (OMC) functionalized by chitosan (OMC-CS) and ferrocene hybrid chitosan (CS) dispersed MWCNTs (Fc@MWCNTs-CS). The aptasensor exhibited a broad linear concentration range from 2.85 × 10^−9^ M to 2.85 × 10^−4^ M with a detection limit of 9.41 × 10^−10^ M. The aptasensor was applied to detect chlorpyrifos in vegetables and fruits samples with good recoveries from 98.5% to 107%. In the second work [104], an aptasensor based on composite film consisting of carbon black (CB) and graphene oxide@Fe_3_O_4_ (GO@Fe_3_O_4_) was developed taking advantage of the high electron transfer ability of CB and GO@Fe_3_O_4_. The proposed aptasensor provided an extremely wide linear range of (2.85 × 10^−10^–2.85 × 10^−4^) M with a detection limit of 9.41 × 10^−11^ M and was applied to monitor chlorpyrifos residues in real vegetable samples.

### 3.2. Optical Aptasensors

**Figure 4:**
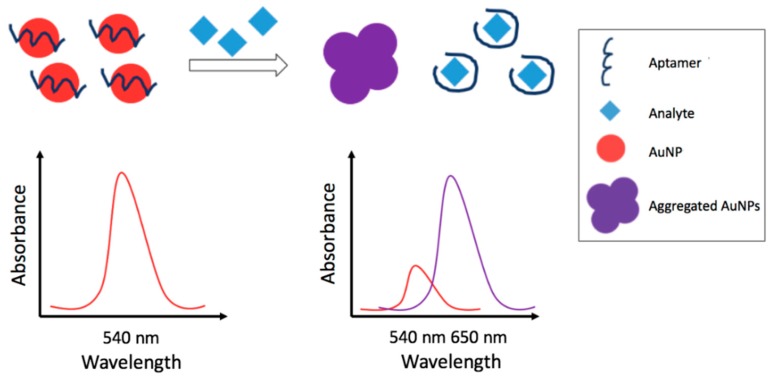
Schematic representation of colorimetric detection strategy employing an aptamer; the strategy is based on the analyte-induced aggregation of AuNPs.

**Figure 5:**
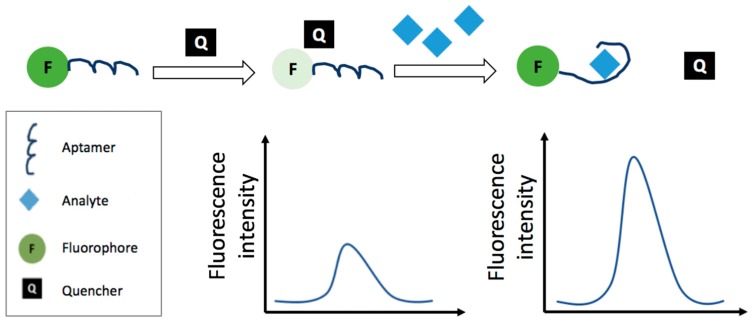
Schematic representation of fluorescence detection strategy employing an aptamer; the strategy is based on the fluorophore fluorescence quenching by the use of a quencher.

Figure 4 displays a colorimetric detection strategy based on the analyte-induced aggregation of AuNPs, while, Figure 5 displays a fluorescent detection strategy based on the quenching of a fluorophore by the use of a quencher. In Table 4 the strategies and features of the optical aptasensors that have been reported in this section have been summarized.

#### 3.2.1. Colorimetric Metal Nanoparticles-Based Aptasensors

In the last years, several authors developed non-competitive colorimetric aptasensors based on the analyte induced aggregation of metal nanoparticles (MNPs), among these, AuNPs and AgNPs were the most used. The aggregation, caused by the analyte-aptamer interaction, leads to changes of the MNPs localized surface plasmon resonance (LSPR) with consequent color/absorbance change. In particular, the MNPs aggregation leads to an LSPR shift that generates a new peak at longer wavelengths and a decrease of the LSPR native peak. In the studies reported, the pesticide quantification is generally carried out reporting the ratio of the absorbance of the new peak (increasing) vs. the native peak (decreasing) after the aggregation. Increase of the amount of pesticide enhances this absorbance ratio. Bala and co-coworkers [105,106,107] proposed different colorimetric aptasensors based strategies for the detection of phorate and malathion based on the aggregation of AuNPs and on the color change from red (absence of pesticide) to blue (AuNPs aggregate in presence of pesticide), easily observable by the naked eye. In one work [105], a simple and rapid method for the selective detection of phorate is reported. In the absence of phorate, the aptamer-modified AuNPs suspension resulted red whereas upon addition of phorate, aggregation leads to the appearance of a blue color. The sensing system had wide linear range, (1.00 × 10^−11^–1.30 × 10^−6^) M, with a limit of detection as low as 1.00 × 10^−11^ M. In order to test the applicability of the method, an analysis of spiked apple samples was performed. Another paper [106] reports the detection of malathion employing an aptamer, a cationic peptide, as aggregation inducer, and unmodified AuNPs. The color of the nanoparticles was red in the absence of malathion since the peptide was bound to the aptamer whereas in the presence of malathion, the aptamer was linked to malathion and the peptide was free and causing aggregation of nanoparticles. A short linear dynamic range from 1.00 × 10^−11^ M to 7.50 × 10^−10^ M was obtained with a low detection limit of 1.94 × 10^−12^ M. A further work [107] reported another detection strategy employing unmodified AuNPs, an aptamer and the positively charged, water-soluble polyelectrolyte polydiallyldimethylammonium chloride (PDDA). PDDA, in this case, bound the aptamer preventing aggregation. Addition of the pesticide, with higher affinity for the aptamer, generated PDDA blue shifting of absorbance. The method was linear in a wide concentration range of (5.00 × 10^−13^–1.00 × 10^−9^) M with an extremely low LOD of 6.00 × 10^−14^ M. Feasibility was tested on apple samples.

Kwon et al. [108] developed a AuNP-based colorimetric multi-aptasensor for the detection of two pesticides, iprobenfos (IBF) and edifenphos (EDI). The AuNPs were stable in presence of aptamers and high salts content, because of protection of the adsorbed poly-negative charge of aptamers on gold surface. Upon addition of the target pesticide, the aptamers were desorbed because of the binding with pesticide and the AuNPs aggregation was obtained. IBF and EDI were detected as low as 1.00 × 10^−11^ M and 5.00 × 10^−9^ M, respectively. This multi-aptasensor was tested in spiked rice samples. For polished and paddy unwashed rice samples, the recoveries were from 81.1% to 104%.

The use of AgNPs have been also reported by Bala et al. [109]. The color of the suspension remained yellow in the absence of malathion due to the electrostatic binding between the aptamer and the cationic peptide which otherwise tends to aggregate AgNPs due to electrostatic interactions. The presence of malathion caused the aggregation of AuNPs turning the suspension to orange. The proposed methodology exhibited a limited linearity range, (1.00 × 10^−11^–7.50 × 10^−10^) M, but a very low LOD of 5.00 × 10^−13^ M. The developed aptasensor was successfully applied to detect malathion in tap water and apple samples, recoveries obtained were in the 89.0–110% range.

#### 3.2.2. Surface-enhanced Raman Scattering-Based Aptasensors

Surface-enhanced Raman Scattering (SERS) is a powerful spectroscopic technique that exploits nanotechnology and Raman spectroscopy. SERS is used for the detection of traces of closely adsorbed molecules on metallic nanostructures (often gold or silver) [117]. The advancement of nanotechnology allowed the production of different nanostructures from nanoparticles to nanowires, that can be used as SERS active substrates [118]. SERS has been exploited by several authors for the label-free detection of pesticides using aptamers as recognition elements. Nie et al. [111] and Barahona et al. [110] reported two aptasensors for the detection of malathion in tap water. Barahona et al. [110] developed a composite material based on polymer-AuNP-aptamer sensing particles capable of acting as capture and signal-enhancer for SERS detection of malathion. Micron-sized polymer particles were synthesized by precipitation polymerization using methacrylic acid and ethylene glycol dimethacrylate as co-monomers. Conjugation with colloidal AuNPs via modification with 2-aminoethanethiol led to polymer-AuNP composites with controlled aggregation of AuNPs onto the polymer surface. The thiolated aptamer targeting malathion was attached to the metal surface by thiol-AuNPs interaction, resulting in a polymer-AuNP-aptamer composite microspheres. The spectra of polymer-AuNPs-aptamer substrate incubated with malathion showed characteristic peaks for the pesticide. The sensor had a limit of detection of 9.99 × 10^−6^ M. In order to test the method, tap water samples were spiked and analyzed. Nie et al. [111] proposed silver nanoparticles modified with positively charged spermine as enhancing and capture reagents for the negatively charged aptamer. The negative phosphate backbone of the aptamer can be combined with the positive spermine by electrostatic interaction. Then, the silver nanoparticles modified by aptamer were used to specifically capture the malathion and the characteristic peak of the binding of aptamer with malathion was measured. The method had a detection limit of 5.00 × 10^−7^ M and the spiked experiments for tap water showed recoveries from 87.4% to 111%.

Pang et al. [112] developed a simple and rapid method able to detect and discriminate four pesticides (isocarbophos, omethoate, phorate and profenofos) using a single aptamer-based capture procedure followed by SERS. The thiolated aptamer (Ap) was conjugated onto silver (Ag) dendrites, a nanostructure that can enhance the Raman fingerprint of pesticides, through Ag-thiol bonds. After capturing the pesticides, the Ag-(Ap + MH)-P complex was analyzed. The results showed that the four pesticides can be captured and discriminated using principal component analysis based on their distinct fingerprint Raman peaks. The limits of detection (LODs) of isocarbophos, omethoate, phorate, and profenofos were 3.40 × 10^−6^ M, 2.40 × 10^−5^ M, 4.00 × 10^−7^ M and 1.40 × 10^−5^ M, respectively. Thus, the LODs were not so satisfactory, except for phorate.

#### 3.2.3. Fluorescence Resonance Energy Transfer-Based Aptasensors

Fluorescence Resonance Energy Transfer (FRET) is an optical technique in which a donor fluorophore is excited by incident light and the excited state energy can be transferred to an acceptor placed at a close distance. This leads to reduction of the donor fluorescence intensity and excited state life time [119]. The system can be used as a switch for affinity sensing. For example, Arvand and Mirroshandel developed a graphene oxide-based FRET sensor for edifenphos detection [113]. Graphene oxide (GO) was used as an acceptor in combination with the aptamer as the recognition element and the ZnS quantum dots (QDs) as donor. QD-aptamer was adsorbed on GO; GO quenched the fluorescence emission intensity of the QDs. In the presence of edifenphos, the QD-aptamer was released from the GO sheets, after the aptamer-pesticide binding, and the fluorescence intensity restored. The intensity resulted proportional to the pesticide concentration with a detection limit of 4.19 × 10^−10^ M and the sensor was applied for monitoring edifenphos in spiked rice samples. Hu et al. [114] and Lin et al. [115] also reported aptasensors based on FRET detection of acetamiprid. Hu et al. [114] proposed an aptamer-based upconversion nanosensor with FRET between NH_2_- NaYF_4_: Yb, Ho@SiO_2_ (upconversion nanoparticles, UCNPs) and AuNPs. AuNPs (functionalized with the aptamer), as acceptors, quenched the fluorescence of UCNPs; when the aptamer interacts with the pesticide leads to the aggregation of AuNPs. The fluorescent intensity of the detection system gradually increased with the increasing concentration of acetamiprid. The limit of detection was 3.20 × 10^−9^ M. Spiked tea samples were analyzed with satisfactory recoveries ranging from 97.6% to 102%. A fluorescence probe (ZnS:Mn-Aptamer) was designed by Lin et al. [115] conjugating ZnS:Mn and QDs functionalized with an acetamiprid binding aptamer. The fluorescence of the probe was turned off by MWCNTs based on FRET between QDS ZnS:Mn-Aptamer and MWCNTs. The fluorescence intensity increased with the increase of the pesticide concentration giving a sensitive “turn-on” based sensor; the detection limit obtained was 7.00 × 10^−10^ M. In order to test the applicability of the sensor, spiked cabbage leaves samples were analyzed and the recoveries obtained were from 90.0% to 95.0%.

Guo et al. [116] reported a label free non-competitive aptamer-based fluorescent method for selective detection of acetamiprid based on the inner filter effect (IFE) of AuNPs on the fluorescence of CdTe quantum dots (CdTe QDs). This approach did not require the link of AuNPs with QDs, and offered considerable flexibility and simplicity. When CdTe QDs were mixed with AuNPs (functionalized with the aptamer), the fluorescence of CdTe QDs was significantly quenched via IFE. However, in the presence of acetamiprid, the aptamer had a conformational change, losing the ability to ‘protect’ AuNPs and causing aggregation. Consequently, the IFE-decreased emission of CdTe QDs was regained. The method had a detection limit of 7.29 × 10^−9^ M. The proposed aptamer-based fluorescent method has been used to assess low amounts of acetamiprid in Chinese cabbage with recoveries from 85.7% to 90.9%.

## 4. Molecularly Imprinted Polymer Sensors

Molecular imprinting technology (MIT) allows the fabrication of molecularly imprinted polymers (MIPs), created to mimic biological receptors [27], by polymerization of a functional monomer in the presence of the analyte (template) and by subsequent extraction of the template by the polymer matrix formed. The imprinting polymerization methods can be divided into bulk polymerization, in situ polymerization (such as electro-, photo- or thermal-polymerization), precipitation polymerization, suspension polymerization, two-step swelling and polymerization, surface imprinting, and sol–gel methods [120]. In all the cases, after the polymerization process, the template is removed from the polymer leaving specific recognition sites complementary in shape, size, and chemical functionality to the template molecule [121,122]. MIPs possess many promising characteristics compared to the biological receptors, such as low cost and ease of synthesis, high stability to harsh chemical and physical conditions and long term reusability [121,122,123]. In the last years several authors have reviewed molecular imprinting polymers [121,122,123,124,125] and their use as recognition element in electrochemical sensors [126,127,128,129,130], optical sensors [129,131], and piezoelectric sensors [129,132,133]. Zhong et al. [134] have also reviewed the use of nanomaterials in molecularly imprinted electrochemical sensing. MIP sensors have been used for the detection of different compounds, including pesticides, and their application for the pesticides detection in food and environment have been recently reviewed [135,136,137]; here we report recent MIP sensors significant applications.

### 4.1. Electrochemical MIP Sensors

**Figure 6:**
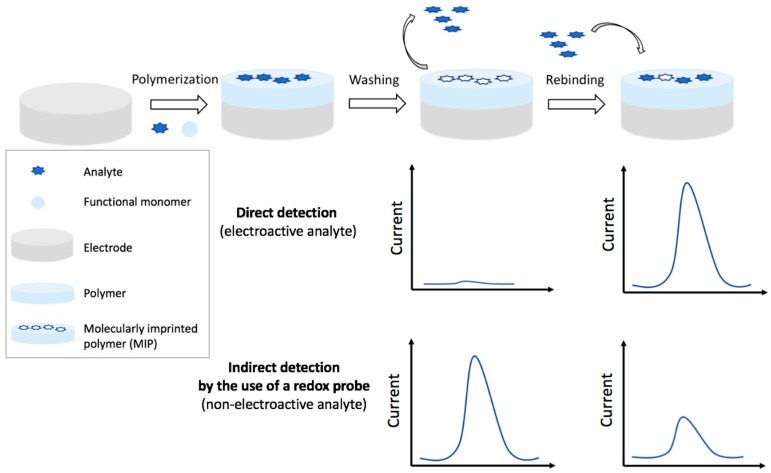
Schematic representation of an electrochemical molecular imprinted polymers (MIP) sensor production and detection of the analyte: direct detection and indirect detection by the use of a redox probe.

The strategies and features of electrochemical MIP sensors discussed in this section are summarized in Table 5. The MIPs can be obtained by different kinds of polymerization, the most used is the electrochemical, since polymer formation, and thickness can be easily controlled. Among the different detection techniques, DPV is the most employed, followed by Linear Sweep Voltammetry (LSV), SWV, potentiometric, CV, and EIS. Indirect detection for non-electroactive analytes is carried out using a redox probe as for antibodies and aptamers, the limitation of the diffusion towards the electrode surface is, in fact, obtained when the target is bound to the polymer. Figure 6 schematizes the production steps of an electrochemical MIP sensor and the related detection strategies.

#### 4.1.1. Voltammetric MIP Sensors

##### Direct Detection

In this section MIP based sensors for the detection of different electroactive pesticides are reported; in this case, the current signal is related to the electroactive pesticide retained by the MIP.

Khadem et al. [158,159] proposed two MIP-carbon paste electrodes (MIP-CPE) based sensors, with similar sensing performances, for the detection of dicloran and diazinon in tap water by SWV. The MIPs were obtained by polymerization of methacrylic acid (MAA). These sensors were used to determine the pesticides in tap water samples with recoveries ranging from 94.2% to 96.5% for dicloran and from 94.0% to 96.5% for diazinon, without particular sample preparation. Another MIP-CPE based sensor obtained by the polymerization of MAA for the detection of diazinon by SWV was developed by Motaharian et al. [160]. They synthesized the MIP nanoparticles (nano-MIP) by suspension polymerization. For preparation of modified CPE, graphite powder was mixed with the nano-MIPs and paraffin oil. The sensor showed a detection limit of 7.90 × 10^−10^ M and was successfully applied for the determination of diazinon in apple fruit samples. Furthermore, the signal of the sensor remained up to 91.8% of its initial value after 14 weeks, suggesting good stability.

Kumar et al. [161] developed a sensor to detect mancozeb (MCZ) by SWV using superparamagnetic iron oxide nanoparticles (SPIONs) coupled to molecularly imprinted star polymers (MISP). The imprinted star polymer-modified SPIONs were synthesized via a surface imprinting approach using itaconic acid as monomer. Recovery of MCZ in spiked vegetable samples were in the 99.0–100% range. After six months of storage, the electrode did not show any change in the current response.

Li et al. [151] fabricated a MIP-based disposable sensor for paraoxon (PO) determination by DPV. The sensor was based on a screen-printed carbon electrode (SPCE) modified with a surface molecularly imprinted poly (*p*-aminothiophenol) (*p*-ATP)/AuNPs composite film, which consisted of a *p*-ATP outer layer and an AuNPs inner layer. The detection limit was 1.00 × 10^−9^ M and the proposed sensor was successfully applied for the determination of PO in cabbage and apple. After three months at 4 °C, the electrode had a 16.0% decrease in the PO signal, indicating an excellent stability.

A MIP film created on a graphene-modified electrode for the selective determination of phoxim by DPV was proposed by Tan et al. [148]. The MIP was produced by a free radical polymerization method using acrylamide (AM) as monomer. The sensor showed a detection limit of 2 × 10^−8^ M and was employed to determine phoxim in cucumber samples with recovery ranging from 98.1% to 101%.

Hu et al. [149] fabricated a simple sensor based on a carbon paste electrode modified with core-shell aluminum doped surface molecularly imprinted siloxane for the sensitive detection of dimetridazole (DMZ) by DPV. Because of aluminum doping, the surface imprinting sensor exhibited higher rebinding capacity, recognition ability and affinity for DMZ, in comparison with the aluminum free sensor. The detection limit obtained was 3.60 × 10^−9^ M and the sensor was successfully employed to detect DMZ in egg and milk powder, with good recoveries ranging from 93.0% to 108%. When stored in air at room temperature, the sensor retained the 95.5% of its initial response after two months.

A MIP sensor with double catalytic effect was reported by Li et al. [147] for carbofuran detection. They developed a MIP sensor based on MWCNT supported Pd-Ir nanocomposite and methylene blue (MB) (as signal amplifier). MWCNT/Pd-Ir composite was synthesized and used to modify the GCE surface, then a MIP was prepared by electropolymerization, with MB-doped *o*-phenylenediamine as functional monomer. Due to the double catalytic effect of MWCNT/Pd-Ir and MB, the DPV current intensity for carbofuran was amplified. The sensor exhibited a very low LOD (1.70 × 10^−12^ M). The method was applied to detect the pesticide in spiked vegetable and fruit samples.

Despite the inherent lower sensitivity of linear sweep voltammetry (LSV) vs. DPV or SWV some authors report the use of LSV coupled to MIP sensors. Xie et al. [152] and Zhang et al. [153] proposed two MIP sensors exploiting the LSV for the detection of pesticides in brown rice. The MIPs were obtained by free-radical polymerization of *p*-vinylbenzoic acid (VBA) on the surface of a GCE modified with graphene (GN). Xie et al. [152] reported a facile synthesis of a MIP sensor for detection of thiamethoxam residue; the detection limit was 4.00 × 10^−8^ M and the recovery range was 88.7–94.0%. Zhang et al. [153] proposed a sensor for the determination of imidacloprid (IMI) residue. The detection limit was 1.00 × 10^−7^ M and the method gave acceptable recoveries from 75.0% to 78.0%. A MIP sensor for the imidacloprid detection by LSV with similar LOD (4.00 × 10^−7^ M) but better recovery in real samples was proposed by Kong et al. [156]. They fabricated a sensor based on imprinted poly(*o*-phenylenediamine) membranes at reduced graphene oxide (rGO) modified GCE. The imprinted electrochemical sensor was successfully employed for the selective determination of imidacloprid in pears samples with recoveries from 91.3% to 96.6%. A decrease in the LOD for the detection of imidacloprid by DPV was obtained by Li et al. [150]. They developed a supramolecular imprinted sensor based on a Pt-In catalytic nanocomposite film and bromophenol blue (BB) amplification. The Pt-In nanocomposite film was electrodeposited on the GCE surface. The composite MIP was prepared by electropolymerization using bromophenol blue doped *o*-aminophenol as functional monomer. A detection limit of 1.20 × 10^−11^ M was obtained. An amplification of the current intensity of IMI was observed, due to the double catalytic effect of Pt-In film and BB. The sensor was successfully used for the analysis of real vegetable samples.

Methyl parathion was detected recently by DPV using two MIP sensors [145,146]. A MIP–ionic liquid–graphene composite film coated GCE (MIP–IL–EGN/GCE) was proposed by Zhao et al. [145]. It was fabricated by coating a GCE with IL-graphene oxide (GO) mixture, followed by the MIP suspension. The MIP was prepared by free radical polymerization using MAA as functional monomer. A detection limit of 6.00 × 10^−9^ M was achieved, and the sensor was applied for the determination of the pesticide in cabbage and apple peel samples with good recovery from 97.0% to 110%. A sensor with improved analytical performances was also reported by He et al. [146]. They developed a sensor based on zinc porphyrin molecularly imprinted polymer microspheres (MIPMs), AuNPs, and carboxyl graphene (CG). The porphyrin zinc-based sensor was fabricated by attaching MIPMs onto the AuNPs/CG nanocomposites. The introduction of AuNPs/CG nanocomposite significantly increased the effective electrode area and amplified the sensor signal. The sensor exhibited a LOD of 3.16 × 10^−10^ M. A MIP sensor for the detection of methyl parathion by LSV with similar analytical performances was reported by Wu et al. [154]. They developed a MIP sensor using nanocomposites by the electrochemical polymerization of *p*-ATP in presence of functionalized AuNPs. The MIP was obtained on the AuNPs decorated MWCNTs modified GCE (AuNPs-MWCNTs/GCE) in presence of AuNPs functionalized with 2-mercaptoethane sulfonic acid and *p*-ATP. The detection limit was 3.04 × 10^−10^ M. This sensor was also applied in the detection of methyl parathion in apples, cucumbers and tap water. Wang et al. [163] developed a MIP sensor based on polyquercetin(Qu)-polyresorcinol(Re)-AuNPs modified GCE for selective methyl parathion determination by CV. The sensor exhibited analytical performances lower than those of the other sensors reported above for the methyl parathion. The detection limit was found to be 1.00 × 10^−8^ M and spiked samples (water, fruit juice, vegetable juice) were analyzed with recoveries ranging from 87.7% to 125%.

##### Indirect Detection

In this paragraph MIP based sensors for the detection of non-electroactive pesticides, and, thus quantified indirectly by the use of a redox probe are reported.

Kong et al. [165] developed a selective MIP sensor for metolcarb (MTMC) detection. The GCE surface was modified with a composite that consisted of polypyrrole (PPy), functionalized MWCNTs and binuclear phthalocyanine cobalt(II) sulfonate (BiCoPc). The composite was, then, modified by the electropolymerization of poly-*o*-aminophenol (PoAP) for the production of the MIP. The pesticide was detected by CV and the detection limit was 7.88 × 10^−9^ M. The sensor was successfully applied to the determination of the MTMC residue in spiked cucumber and cabbage samples with recoveries ranging from 88.8% to 93.3%. Improved analytical performances for the detection of metolcarb were obtained by Yang et al. [157]. They developed a three-dimensional (3D) molecularly imprinted electrochemical sensor (MIECS) for ultrasensitive and specific quantification of metolcarb by LSV. The sensor was based on Prussian blue (PB) mediated amplification combined with signal enhancement of ordered mesoporous carbon material (CMK-3). The MIPs were synthesized by electropolymerization using para aminobenzoic acid (*p*-ABA) as monomer. CMK-3 was introduced to enhance the electrochemical response by improving the structure of the modified electrodes and facilitating charge transfer processes of PB which was used as redox probe. The sensor offered an excellent current response in the linear range of (5.00 × 10^−10^–1.00 × 10^−4^) M and the limit of detection was calculated to be 9.30 × 10^−11^ M. The sensor has been successfully applied for the determination of metolcarb in real samples (cucumber, cabbage, and apple juice) with good recoveries from 92.4% to 98.6%.

Zhao et al. [141] developed a MIP sensor for the DPV determination of carbaryl. GCE was coated with chitosan-AuPt alloy nanoparticles (CS-AuPtNPs) and graphene-ionic liquid-nano Au (GR-IL-Au). Electrodeposition of carbaryl imprinted poly(*p*-aminothiophenol) (*p*-ATP) film was the performed. The CS-AuPtNPs and GR-IL-Au composites played the roles to immobilize the *p*-ATP monomer and improve the electrochemical response. The detection limit was 8.00 × 10^−9^ M. The sensor was applied to the determination of carbaryl in cabbage and apple peel samples.

Our group [162] developed a screening method for the selective and sensitive detection of dimethoate residues in wheat flour by coupling a MIP sensor to a microextraction by packed sorbent (MEPS) strategy. The method consists of MEPS that allows the analyte extraction and preconcentration/clean up, followed by MIP-GCE detection by SWV. The MIP films were electropolymerized onto a GCE, with pyrrole (Py) serving as monomer. The MEPS/MIP procedure was applied to flour samples spiked with dimethoate and data obtained were comparable with a validated UHPLC-MS/MS procedure.

Li et al. [164] developed a highly sensitive sensor for the detection of methyl parathion by CV using a CPE modified with surface molecularly imprinted polymeric microspheres (SMIPMs). Molecular imprinting technique based on distillation precipitation polymerization was applied to prepare SMIPMs using MAA as functional monomer. SMIPMs/CPE exhibited a high sensing response towards methyl parathion with an excellent detection limit of 3.40 × 10^−13^ M. The sensor was used to determine the pesticide in vegetable samples; recoveries ranged from 97.2% to 101%.

A MIP sensor for the detection of carbofuran by DPV based on GCE decorated by reduced graphene oxide and AuNPs (rGO@Au) was fabricated by Tan et al. [140]. The MIPs were prepared on the electrode surface with MAA as functional monomer. The rGO@Au NPs were introduced into the imprinted membrane to improve the electrochemical signal and recognition capacity of the sensor. The sensor had a detection limit of 2 × 10^−8^ M and was successfully applied to the detection of carbofuran in real vegetable samples with a recovery range of 97.7–111%.

A MIP sensor for the detection of glyphosate (Gly) uisng DPV was developed by Xu et al. [144]. They fabricated a selective MIPs device using prussian blue (PB) combined with signal enhancement of urchin-like AuNPs. PB particles electrodeposited on the electrode were used as quantitative electrochemical mediator. The MIP membrane was electropolymerized on the surface of the Au-PB composites modified ITO electrode using pyrrole as monomer. The response was linear in a short range, (2.37 × 10^−6^–7.10 × 10^−6^) M, and the limit of detection was 5.44 × 10^−7^ M. The method was successfully applied to the determination of glyphosate in spiked corn samples with good recoveries from 97.5% to 101%. A simple sensor for Gly detection was prepared by Zhang et al. [139]. They developed a sensor via synthesis of MIPs on a gold electrode by electropolymerization of Py. The detection limit was in this case 1.60 × 10^−9^ M. The sensor was used to detect the concentration of Gly in cucumber and tap water samples. An excellent LOD for Gly was obtained by Do et al. [155]. They developed a sensitive electrochemical sensor based on molecularly imprinted metal-organic frameworks (MOFs). The MIP sensor was obtained by the electrochemical polymerization of *p*-ATP in presence of functionalized AuNPs (FuAuNP). MOFs are crystalline porous hybrid materials comprising coupling units (metal ions or metal–oxo units) coordinated by electron-donating organic ligands. MIP-MOF films were prepared on gold surfaces by electropolymerization of *p*-ATP functionalized AuNPs. This sensor allowed an indirect electrochemical detection of Gly using a non-catalytic electrode material. A linear relationship was obtained in an extremely wide concentration range, (5.90 × 10^−15^–5.90 × 10^−9^) M, with a very low limit of detection of 5.00 × 10^−15^ M. The developed sensor was successfully applied to detect Gly in tap water samples.

Wang et al. [142] built a MIP sensor based on amino-functionalized silica nanoparticles for the detection of 2,4-dichlorophenoxyacetic acid (2,4-D) using DPV. The MIP was prepared on the hierarchical porous dendrimer-like silica nanoparticles (HPSNs-NH_2_) modified electrode via electropolymerization by using *o*-PD as monomer. The porous structure of HPSNs-NH_2_ reduced the diffusion limitations of the analytes, enhanced the accessibility and increased the surface area of the sensor. The detection limit was down to 1.17 × 10^−11^ M and this method was applied to detect 2,4-D in bean sprout samples with satisfactory recoveries ranging from 94.4% to 108%.

Duan et al. [138] constructed a simple MIP-GCE for acephate detection using electropolymerization of *o*-PD; a detection limit of 1.30 × 10^−7^ M was obtained. Spiked tea soup samples were analyzed with recoveries from 96.8% to 104%. Tang et al. [143] described a sensor for the rapid detection of acephate and trichlorfon. The sensor was modified with Fe_3_O_4_@carboxyl-functionalized MWCNTs/chitosan nanocomposite layer (Fe_3_O_4_@MWCNTs-COOH/CS) using molecularly imprinted film as recognition element. The bare GCE was coated with Fe_3_O_4_@MWNTs-COOH/CS and the MIP film was prepared by a sol-gel technology using 3-aminopropyltriethoxysilane (APTES) as functional monomer. The imprinted sensor had very broad linear current responses to acephate and trichlorfon concentrations in the ranges from 1.00 × 10^−10^ M to 1.00 × 10^−4^ M and from 1.00 × 10^−11^ M to 1.00 × 10^−5^ M, respectively. The imprinted sensor limits of the detection result were 6.81 × 10^−11^ M for acephate and 8.94 × 10^−12^ M for trichlorfon. The developed device was successfully applied to detect the pesticides spiked in fortified kidney bean and cucumber samples with recoveries ranging from 85.7% to 94.9%.

#### 4.1.2. Potentiometric and Impedimetric MIP Sensors

Anirudhan and Alexander [166,167] synthesized MWCNT based imprinted polymer (MIP/MWCNT), using MAA as monomer for the direct quantification of 2,4-D and lindane. The applied potential varied with the increase of the pesticide concentration. This was because the lindane itself acted as an electroactive species varying the potential of the cell at zero current flow. A MIP/MWCNT was synthesized on the surface of Cu electrode [166], the sensor gave a linear calibration in the wide range of (1.00 × 10^−9^–1.00 × 10^−3^) M with a LOD of 1.00 × 10^−10^ M. The sensor was used to detect lindane spiked in different samples (tap water, orange, grape, tomato, and cabbage). The same authors [167] prepared a potentiometric sensor based on ion imprinted polymer inclusion membrane (IPIM) produced modifying a MWCNT based molecularly imprinted polymer for the trace determination of the pesticide 2,4-D. The sensing membrane was developed by the inclusion of 2,4-D imprinted polymer materials in polyvinyl chloride (PVC) matrix. The IPIM sensor responded in the range of (1.00 × 10^−9^–1.00 × 10^−5^) M; the detection limit was found to be 1.20 × 10^−9^ M. The stability of MWCNT/IPIM sensor was found to be three months and could be reused more than 30 times with no loss in sensitivity. Experiments with spiked tap water were carried out and good recoveries, from 97.6% to 99.2%, were obtained.

Abdel-Ghany et al. [168] developed different MIP sensors for the detection of dinotefuran: four of these sensors were based on a newly designed MIP material consisting of acrylamide or methacrylic acid as functional monomer in a plasticized PVC (polyvinyl chloride) membrane. The sensors were applied for the determination of the dinotefuran in spiked cucumber samples with recoveries in the range of 87.9–106%. MIP/pencil graphite electrodes (MIP/PGE) obtained by electrochemical polymerization of pyrrole were reported by Uygun and Dilgin [169] and Prusty and Bhand [170]. The first synthesized an impedimetric sensor for chlorpyrifos (CPF); the detection limit obtained was 1.28 × 10^−8^ M. The fabricated sensor was successfully applied to determine CPF in spiked tap water and corn leaves samples with recoveries varying from 101% to 103%. Prusty and Bhand developed a capacitive sensor for 2,4-D determination in drinking water with a limit of detection of 9.05 × 10^−11^ M. Spiked tap and drinking water samples were analyzed with high recovery from 92.0% to 110%.

### 4.2. Optical MIP Sensors

**Figure 7:**
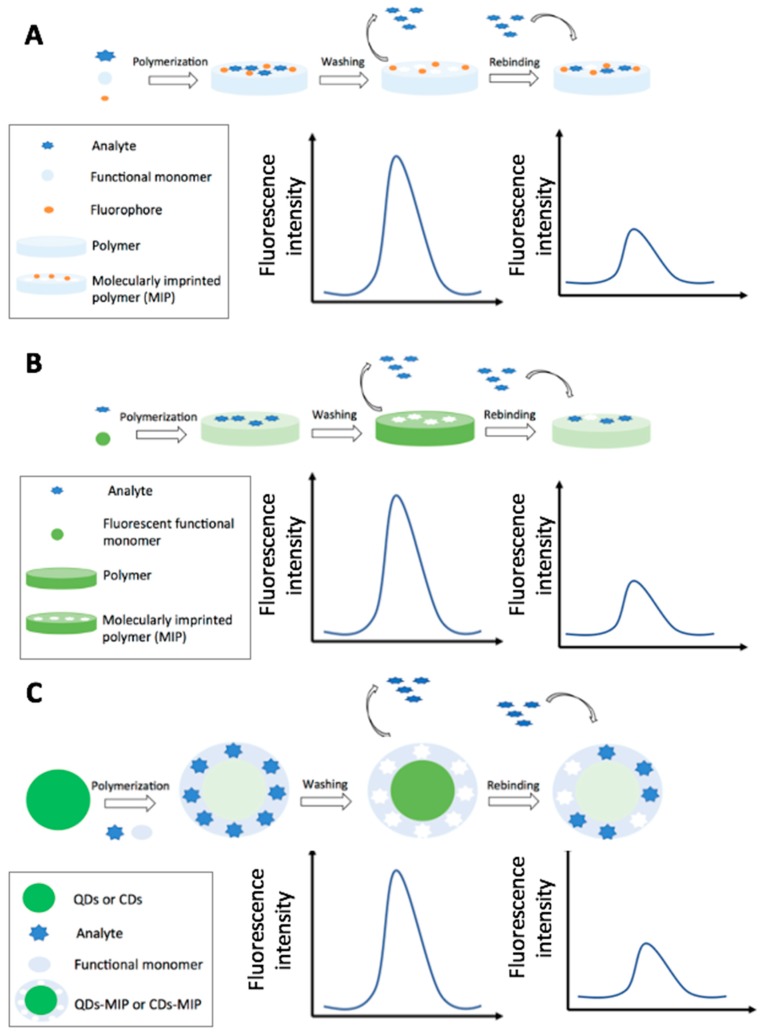
Schematic representation of optical MIPs production with the main fluorescence-based detection strategies by the use of a fluorophore (**A**); by the use of a fluorescent functional monomer (**B**) and exploiting the fluorescence quenching of QDs or CDs (**C**).

Figure 7 displays the production steps for optical MIPs and a schematization of the main fluorescence-based detection strategies. Table 6 summarizes the strategies and features of the optical MIP sensors reported in this section.

#### 4.2.1. Fluorescence and Fluorescence Resonance Energy Transfer-Based MIP Sensors

Fluorescence has been historically the most employed optical transduction strategy for the MIPs sensors used for pesticides detection in food. The detection has been carried out using the quenching effect on fluorescent probes, as quantum dots (QDs), carbon dots (CDs), other fluorophores or by FRET.

The development of MIP synthesized by the polymerization of MAA and based on the quenching of the fluorescence of CdSe/ZnS QDs for the detection of pesticides was reported by several authors. Liu et al. [171] designed and synthesized a MIP fluorescent probe based on CdSe/ZnS QDs (CdSe/ZnS@MIP) obtained by bulk polymerization, for the detection of methamidophos. The pesticide had a good fluorescent quenching effect to CdSe/ZnS@MIP. The method showed a limit of detection of 9.16 × 10^−8^ M. Spiked apple and pear samples were analyzed with good recoveries from 89.7% to 94.9%. Zhou et al. [172] fabricated a QDs based-MIP as fluorescent probe for the detection of carbofuran. The MIP was synthesized by a multi-step swelling and polymerization method, and, then, labeled with CdSe/ZnS QDs via a gradual solvent evaporation method. The fluorescence intensity of the QDs-MIP decreased linearly with the increase of carbofuran concentration exhibiting a LOD of 9.04 × 10^−10^ M. This MIP was applied for carbofuran detection in tap water samples. Zhang et al. [173] developed a fluorescent sensing material based on CdSe/ZnS QDs and MIP for the detection of carbaryl. The surface of CdSe/ZnS QDs was modified with ionic liquids (ILs) by electrostatic interaction. The fluorescent sensing material was built anchoring the MIP layer on IL modified CdSe/ZnS QDs by copolymerization. The LOD obtained was 1.47 × 10^−7^ M and the MIP was successfully applied to analyze carbaryl in rice and Chinese cabbage samples. Acceptable recoveries ranging from 74.0% to 88.0% were obtained.

Fluorescent MIP synthesized by polymerization of APTES and based on the fluorescence quenching of CdTe QDs were proposed by Jia et al. [174] and Tang and Xiang [175]. Mesoporous structured imprinted microspheres on the surfaces of CdTe QDs for the detection of 2,4-D was proposed in the first work. In the presence of 2,4-D, the fluorescence intensity of the sensor was weakened because of electron transfer. The sensor exhibited a detection limit of 2.10 × 10^−9^ M. The sensor still provided nearly equivalent performances after storage at 4 ◦C for two months. Tang and Xiang [175] prepared MIP capped CdTe QDs for the detection of parathion by surface molecularly imprinting CdTe nanoparticles with molecular recognitive activity using reverse microemulsion polymerization; APTES was the functional monomer. The sensor showed an extremely wide detection range, (5.00 × 10^−8^–1.00 × 10^−3^) M, with a detection limit of 2.18 × 10^−7^ M. This method was used successfully to detect parathion in tap water with recoveries ranging from 99.3% to 100%.

Wang et al. [176] used MIPs and ZnO QDs for the determination of pyrethroids. Monodisperse ZnO quantum dots were prepared by a sol-gel process and modified by 3-(methacryloyloxy)propyl trimethoxysilane. The ZnO-based MIPs were employed for the determination of cyhalothrin in milk with recoveries ranging from 99.6% to 103%. Li et al. [177] synthesized a molecularly imprinted silica layer appended to FeSe QDs (MIP-FeSe-QDs) as a recognition element, by modified reverse micro-emulsion, using APTES and MAA as functional monomers, for cyfluthrin determination. After the specific recognition of MIP-FeSe-QDs to cyfluthrin, the charge transfer from the FeSe-QDs to cyfluthrin was blocked and resulted in the fluorescence quenching of the MIP-FeSe-QDs. The exploitability of the developed sensor method for the pesticide detection in fish samples was validated and recoveries ranging from 88.0% to 90.7% were obtained.

Another fluorescent probe widely used by several authors for the detection of pesticides is allyl fluorescein. Gao’s group [178,179,180,181] reported the detection of cyhalothrin and λ-cyhalothrin in honey and soda water using MIPs obtained by polymerization of acrylamide (AM) in presence of allyl fluorescein. Ultra-trace cyhalothrin was detected in honey samples by the use of fluorescent MIP microspheres obtained via precipitation polymerization [178] and by the use of fluorescent SiO_2_@KH570-MIP [179] obtained by the surface-imprinting technique. The LOD obtained was very low (4.00 × 10^−12^ M), with recoveries ranging from 97.0% to 104% [178] and from 94.0% to 114% [179]. The same author has developed a fluorescent core-shell MIP based on SiO_2_ beads (SiO_2_@FMIP) [180] by copolymerization of acrylamide in which the fluorescence was due to the shell. The method (LOD = 5.00 × 10^−12^ M) was satisfactory for determination of λ-cyhalothrin in soda water samples with recovery ranging from 96.0% to 111%. Gao proposed also a core-shell magnetic Fe_3_O_4_/SiO_2_-MPS/MIPs (MPS = 3-(methacryloxyl) propyl trimethoxysilane) obtained by surface molecular imprinting technique, with Fe_3_O_4_/SiO_2_-MPS as core and MIPs as shell [181]. The fluorescent molecularly imprinted polymer shell was firstly prepared by the copolymerization of acrylamide. The LOD was 5.11 × 10^−9^ M and the method was satisfactory used for determination of λ-cyhalothrin trace in honey samples.

Wang et al. [182] synthesized a core-shell fluorescent MIP (SiO_2_@FITC-APTES@MIPs) via fluorescein 5(6)-isothiocyanate (FITC) and APTES/SiO_2_ particles, for the analysis of λ-cyhalothrin. The limit of detection was 9.17 × 10^−9^ M and the sensor was tested for determination of λ-cyhalothrin in tap water and Chinese spirits samples. Li et al. [183] developed a fluorescence switch sensor for detection of the fungicide fenaminosulf (FM), based on a dye-doped MIP and a silver nanofilm. The MIP was prepared by electropolymerization of hydroquinone doped with neutral red on the silver nanofilm modified electrode. A fluorescence signal was produced by the neutral red and the fluorescence intensity was decreased with the increase of pesticide concentration. Therefore, elution and adsorption of FM by the MIP acted as a switch to control the fluorescence intensity, which was amplified by the silver nanofilm. The detection limit obtained was 1.60 × 10^−11^ M, this method was utilized to determine residual FM in vegetable samples with recoveries ranging from 92.0% to 110%.

Some authors used a fluorophore as functional monomer for the production of MIP. In this way, binding events produce fluorescence signals variation. Thus, the pesticides can be detected directly without the use of labels. For example, Li et al. [184] developed a fluorescent MIP sensor for the rapid sensing of alachlor by precipitation polymerization. 2-Acrylamide-6-methoxybenzothiazole (AMMB) was used both as fluorescent reporter and functional monomer. The proposed method was successfully applied for the determination of trace alachlor in corn seeds, with recoveries ranging from 95.6% to 104%. The fluorescent intensity remained almost unchanged at room temperature for 7 weeks, demonstrating the stability of the AMMB-based MIP. Ren et al. [185] used a fluorescent functional monomer (7-allyloxy coumarin) for the synthesis of a MIP by surface molecular imprinting technique for the detection of 2,4,6-trichlorophenol (2,4,6-TCP). The fluorescence intensity of SiO_2_@dye-FMIPs decreased with increasing 2,4,6-TCP concentration. The sensor had a detection limit of 5.34 × 10^−11^ M and was applied to detect the pesticide spiked in soda water samples.

A competitive fluorescent method for the determination of atrazine in tap water was presented by Liu et al. [186]. They developed a magnetic molecularly imprinted polymer (MMIP) for pesticide detection based on competition between atrazine and 5-(4,6-dichlorotriazinyl) aminofluorescein (5-DTAF) (a commercially available fluorescent analog of atrazine), to recognize the specific binding sites. The MMIP, based on Fe_3_O_4_-chitosan nanoparticles, was prepared via the copolymerization of the functional monomer MAA in the presence of atrazine. A detection limit of 8.60 × 10^−7^ M was found and tap water samples were analyzed with recoveries ranging from 77.6% to 115%. The design of this fluorescent assay based on MMIP is simpler than currently proposed MIP fluorescent sensors (i.e., MIP-capped QDs), obviating the need to embed organic fluorescent materials or QDs into the MIPs.

Li et al. [187] developed a MIP fluorescent sensor for competitive dimethoate detection based on fluorescence resonance energy transfer (FRET). The MIP was prepared by electropolymerization on ITO and methyl red (MR)-doped *o*-phenylenediamine, used as functional monomer. Dimethoate in the sample was detected through a competitive MIP binding reaction with carbon dots (CDs)-labeled dimethoate. The detected limit was 1.83 × 10^−11^ M and the sensor was used to detect dimethoate in vegetable samples with recoveries ranging from 95.0% to 106%.

#### 4.2.2. Colorimetric, Dual Read-Out (Fluorescence-Based + Colorimetric) and Electrochromic MIP Sensors

A metabolite of pyrethroid class, 3-phenoxybenzaldehyde (3-PBD), can be oxidized to 3-phenoxybenzoic acid leading to color fading of potassium permanganate. Inspired by this phenomenon, Ye et al. [188] developed a MIP for the detection of 3-PBD. The MIP layer was coated on the surface of monodispersed silica nanoparticles via sol-gel process with APTES and phenyltrimethoxysilane (PTES) as monomers. The obtained detection limit was 2.62 × 10^−7^ M and this method was used to detect 3-PBD in fruit juice and beverage samples with good recovery in the range of 90.0–97.8%.

Zhang et al. [189] proposed a molecularly imprinted membrane-zinc porphyrin-methacrylate (MIM-Zn-MAA), dual read-out sensor (fluorescence + colorimetry) based on a molecularly imprinted membrane, to detect dimethyl methylphosphonate (DMMP), an intermediate molecule of organophosphorus pesticides. The membranes were prepared via thermal polymerization of two functional monomers (zinc porphyrin and methacrylate) on the surface of a glass slide. The pesticide detection was carried out by measuring the difference in the fluorescence intensities before and after the reaction between MIMs (MIM-Zn-MAA) and DMMP. The reaction was followed also by investigating the color variance of MIMs toward DMMP at different concentrations. The fluorescence and colorimetric intensities of the membranes increased as the concentration of DMMP increased. This change was attributed to the interaction between zinc porphyrin and DMMP. The fluorescence intensity and the maps (colorimetric response) showed an extremely wide linear range varying from 1.00 × 10^−7^ M to 1.00 × 10^−2^ M, with a detection limit of 1.00 × 10^−7^ M. Different concentrations of DMMP were spiked into tap water samples and were analyzed by fluorescence spectroscopy with recoveries ranging from 96.5% to 106%.

Recently, a novel electrochromic MIP sensor for the visual and smartphone-based detection of chlorpyrifos was developed by Capoferri et al. [190]. The detection strategy is based on the electrochromic properties of iridium oxide NPs (IrOx NPs) that were exploited, for the first time, to detect an analyte by the use of a MIP sensor. The IrOx NPs are transparent in the reduced state and turn blue-black upon oxidation. The sensor was fabricated using screen-printing technology with indium tin oxide (ITO) as transparent working electrode. Two different approaches were used to detect and quantify the pesticide: direct visual detection and smartphone imaging. In fact, the application of different oxidation potentials for 10 s resulted in visual color changes directly related to the concentration of the analyte. On the other hand, at fixed potential, the concentration of the analyte was dependent on the color intensity of the electrode measured by the use of a smartphone. The electrochromic sensor detected chlorpyrifos quickly in an extremely wide dynamic range, (1.00 × 10^−13^–1.00 × 10^−3^) M, with an excellent detection limit (1.00 × 10^−13^ M). In addition, the sensor was applied to analyze spiked drinking water with recovery values from 81.0% to 107%.

#### 4.2.3. Surface Plasmon Resonance-Based MIP Sensors

Yao et al. [191] proposed magnetic MIPs for SPR detection of chlorpyrifos (CPF). The core-shell imprinted Fe_3_O_4_@polydopamine nanoparticles (Fe_3_O_4_@PDA NPs) were prepared by self-polymerization of dopamine in the presence of CPF on the Fe_3_O_4_ NPs surface. The obtained imprinted Fe_3_O_4_@PDA NPs showed excellent magnetic properties (thanks to the Fe_3_O_4_ core), allowing the direct capture, concentration, and separation of targets in complex samples, simply using a magnet. The detection of CPF was achieved by employing the imprinted Fe_3_O_4_@PDA, which acts both as amplifier to increase the SPR signal then as a special recognition element able to improve the selectivity. The SPR angle shifts resulting from the binding of the imprinted Fe_3_O_4_@ PDA NPs gradually increased with the increase of CPF concentrations. The sensor showed a good linear relationship between the SPR angle shift and the chlorpyrifos concentration over a wide concentration range from 1.00 × 10^−9^ M to 1.00 × 10^−5^ M, showing a detection limit of 7.60 × 10^−10^ M. The proposed device was applied for detecting pesticide residues in spiked apple samples with recoveries ranging from 93.0% to 104%.

## 5. Conclusions

The detection of pesticide residues in food is one of the main concerns in the chemical safety of food considering toxicity and unknown effect of metabolites for the majority of these compounds. There is a considerable effort by regulatory bodies in updating guidelines for sampling, sample treatment and assay methods for the quantification of pesticides, both for compounds having established MRLs or without MRLs. This is particularly true for confirmatory methods that are used by official control labs. The latter, in fact, are expected to play a key role in the control of newly used pesticides in food and in the assessment of the risk given by the presence of active principles or metabolites to human health and to the environment in broad sense. However, it is not reasonable to think that confirmatory methods, mainly based on chromatographic techniques coupled with mass spectrometric detection, can be massively used to assay a very high number of samples ensuring a safe control of the food we eat. In fact, considering the complexity and the cost of the analysis, this does not appear sustainable at all, at least in the short and medium run.

It is extremely important, thus, to have devices able to detect these compounds easily, in a short time, with low costs and without complex sample pretreatment. In principle, these screening devices may be used in decentralized labs or directly in field, to reduce the amount of samples needed for official control (e.g., avoiding to test negative samples). Moreover, they can be used by producers to monitor the amount of pesticide delivered, present on a crop or ready for the market, saving money (lower amount of pesticide used), and releasing a safer product. The same concept applies for the whole food industry chain until retailers and, possibly, consumers.

We think affinity sensors can be appropriate screening tools for filling this gap. The present review gives an overview of recent affinity sensing strategies for electrochemical and optical detection of pesticides in food. Examples on the use of antibodies, aptamers, and molecularly imprinted polymers (MIPs) as recognition elements have been reported, the sensing strategy for the development of the assay are shortly described together with the analytical behavior of the sensors. Most of these affinity sensors satisfy the minimum requirements for the rapid screening of pesticides, particularly detecting the analytes at levels below MRLs set by regulatory agencies.

Detection limits at the picomolar level are reported for a lot of the developed assay and the described sensors show that the combination of novel transduction materials and strategies with improved recognition element can push toward lower and lower achievable detection limits. Is this really necessary in this field? In our opinion it is so. In fact, extraction of the analyte from the sample and matrix effect can play a crucial role in the performance of a sensor based screening assay. Both phenomena can be much more easily tackled in a very sensitive assay by dilution of the sample. Selectivity vs. other similar analytes, in this assay, appears not so important, in our opinion. This type of assay can be used, in fact, for the detection of one molecule, or few molecules, in case of multiplexed formats. Thus, the ideal use of the sensors would be in the monitoring or assessment of the level of the pesticide (or few pesticides) used, or expected to be used, on food commodities.

Taking into consideration the reported categories of affinity sensors we should say that the affinity of the recognition element, as expected, plays a key role for the analytical features of the assay; however, the detection technique (e.g., voltammetry vs. fluorescence etc.) and the assay format (e.g., competitive, non-competitive) can be equally important. For these reasons making a comparison among different approaches to establish, which is the best to develop a screening assay based on affinity sensors, is very difficult. We think, however, that by the examples reported an appropriate format for an affinity sensor for any pesticide can be designed, independently on physicochemical characteristics of the molecules, and on the concentration levels needed to detect.

Some general considerations can be anyway taken. The use of antibodies in affinity sensors as recognition elements will continue to be very relevant only for cases in which there is a clear advantage in affinity vs. aptamers. Aptamers are, in fact, easier to be immobilized with proper orientation on a surface (since are lower in size); moreover, their rearrangement upon binding to the target can give more opportunities to design a sensitive assay. MIP are, in principle, the ideal choice and represent the most intriguing way of designing an affinity sensor; however, the procedures for the synthesis and control of very thin MIP films used in sensors have been developed very recently and need still to be further studied. The use of nanomaterials in the sensors format improved a lot the performance of the assay increasing the signal and also provided the opportunity to immobilize higher amounts of the recognition element (Ab or aptamer) and/or bring the recognition sites closer to the sensing surface (MIPs and nanocomposites sensors). Among the detection techniques, the voltammetric, impedimetric and fluorescent (using a label or FRET) approaches are the most sensitive and well established. Therefore, portable readers are already present in the market. Particularly fascinating is any electrochemical approach measuring the reduced electron transfer of a redox probe, since this does not require any label and the chemistry to obtain the sensor is very simple.

Affinity sensors appear to be very promising to screen pesticides in field; what is still missing is the validation of the assays, including the pretreatment of the samples. Particular attention should be paid, in our opinion, to simple microextraction techniques to be used on site with a reduced amount of solvents. Another key aspect not very often reported in the literature, is long term stability of the sensors and, possibly, reusability. We are anyhow expecting, in the near future, that affinity sensors will be one of the major options for the rapid detection of pesticides in food.

## Figures and Tables

**Table 1:** Electrochemical immunosensors for pesticides detection in food: main features and strategies.

Electrode	Sensing Technique and Redox Probe *	Analyte	Matrix	Linear Dynamic Range LDR (M)	Limit of Detection LOD (M)	Recovery (%)	Ref.
**OVA/Ab/GA/CS/GCE**	EIS	Fenvalerate	Tea	2.38 × 10^−9^–2.38 × 10^−7^	1.91 × 10^−9^	103.0	[58]
**gelatin/Ab/GA/L-Cys/Au electrode**	EIS	Carbofuran	Tomato, cabbage, and lettuce	4.52 × 10^−10^–4.52 × 10^−6^	4.52 × 10^−10^	90.0–106	[59]
**Ab/protein A/AuNPs/PDDA/gold IDAMs**	EIS	Chlorpyrifos	Cucumber, lettuce, and pakchoi	1.43 × 10^−9^–1.43 × 10^−6^	1.43 × 10^−9^	75.2–96.5	[60]
**BSA/Ab/protein A/gold IDAMs**	EIS	Chlorpyrifos	Chinese chives, lettuce, and cabbage	2.85 × 10^−9^–2.85 × 10^−4^	3.99 × 10^−11^	87.6–96.5	[61]
**Ab/AuNPs-PANABA-MWCNTs/SPE**	EIS	2,4-D	Tap water	4.52 × 10^−9^–4.52 × 10^−7^	1.36 × 10^−9^	82.0–120	[62]
**Ab/fG/SPE**	EIS	Parathion	Tomato and carrot	3.43 × 10^−13^–3.43 × 10^−9^	1.79 × 10^−13^		[63]
**BSA/Ab/AuNPs/Au**	DPV	Atrazina	Maize	2.32 × 10^−10^–2.32 × 10^−9^	7.42 × 10^−11^	95.5–120	[64]
**BSA/Ab/AuNPs/PANI/MWCNTs-CS/GCE**	CV	Chlorpyrifos	Cabbage, pakchoi, lettuce, and leek	2.85 × 10^−10^–1.14 × 10^−7^ 1.14 × 10^−7^–1.43 × 10^−6^	1.71 × 10^−10^	80.6–109	[65]
**BSA/Ab/GS-MB/AuNPs/GCE**	CV	Chlorpyrifos	Cabbage, pakchoi, lettuce, and leek	2.85 × 10^−9^–1.43 × 10^−6^	1.60 × 10^−10^	86.0–105	[66]
**BSA/AuNPs-Ab/GS-PEI-Au/MWCNTs/GCE**	CV	Carbofuran	Lettuce, cabbage, green peppers, tomatoes, Chinese chives, and peaches	2.26 × 10^−9^–2.26 × 10^−6^	1.36 × 10^−10^	86.0–103	[67]
**ATR-BSA/PAMAM/AET/Au/GCE**	CV	Atrazine	Corn flakes	4.64 × 10^−11^–4.64 × 10^−6^	5.56 × 10^−9^	109–114	[68]
**BSA/Antigen/Co_3_O_4_/PANI/ITO**	CV	Chlorpyrifos	Green vegetables and apples	0–2.85 × 10^−5^	2.85 × 10^−8^	82.8–107	[69]
**SWNTs modified GCE**	SWV (FDMA)	Paraoxon	Tap water Purified water	7.27 × 10^−9^–9.08 × 10^−6^	7.27 × 10^−9^	92.0 (tap water) 95.0 (purified water)	[71]
**SWNTs/GCE**	SWV (FDMA and PQQ)	Endosulfan and paraoxon simultaneous detection	Tap water Purified water	1.23 × 10^−10^–2.46 × 10^−7^ (endosulfan) 7.27 × 10^−9^–9.08 × 10^−6^ (paraoxon)	1.23 × 10^−10^ (endosulfan) 7.27 × 10^−9^ (paraoxon)	95.0–96.0 97.0–98.0	[70]
**GEC**	SWV (No redox probe)	Paraquat	Potato	1.20 × 10^−8^–2.63 × 10^−7^	3.11 × 10^−11^	76.0–97.0	[72]
**m-GEC**	Amperometry (No redox probe)	Paraquat	Potato		7.00 × 10^−10^	91.8–144	[73]

* When not specified, the redox probe used is the ferrocyanide/ferricyanide redox couple. OVA: ovalbumine; GA: glutaraldehyde; CS: chitosan; GCE: glassy carbon electrode; L-cys: L-cysteine; Au: gold; AuNPs: gold nanoparticles; PDDA: poly (diallydimethylammonium chloride); IDAMs: interdigitated array microelectrodes; BSA: bovine serum albumin; PANABA: polymer poly-(aniline-*co*-3-aminobenzoic acid); MWCNTs: multi-walled carbon nanotubes; SPE: screen-printed electrode; fG: graphene sheets functionalized; PANI: polyaniline; GS-MB: graphene sheets-methylen blue; GS-PEI: graphene sheets-ethyleneimine polymer; ATR: atrazine; PAMAM: poliamidoaminic dendrimers; AET: 2-aminoethanethiol; ITO: indium tin oxide; SWNTs: single wall nanotubes; GEC: graphite composite electrode; m-GEC: magnetic graphite–epoxy composite electrode; FDMA; ferrocenedimethylamine; PQQ: pyrroloquinoline quinone; 2,4-D: 2,4-dichlorophenoxyacetic acid.

**Table 2:** Optical immunosensors for pesticides detection in food: main features and strategies.

Sensing Technique	Analyte	Matrix	Linear Dynamic Range LDR (M)	Limit of Detection LOD (M)	Recovery (%)	Ref.
**SPR**	Triazine	Bovine milk	1.00 × 10^−7^–1.50 × 10^−6^ (atrazine)	5.30 × 10^−8^ (atrazine)	94.9–103 (atrazine)	[74]
					92.6–95.8 (triazine)	
**SPR**	Triazophos	Chinese cabbage, cucumber, and apple	3.13 × 10^−9^–2.65 × 10^−8^	3.06 × 10^−10^	84.4–109	[75]
**SPR**	Fungicide Boscalid	Cucumber, tomato, green sweet pepper, cabbage, spinach, and orange	4.95 × 10^−8^–2.33 × 10^−7^		85.0–109	[76]
**SPR**	Fungicide Chlorothalonil	Lettuce, cabbage, and long green onion	3.01 × 10^−8^–1.65 × 10^−7^		90.0–118	[77]
**Fluorescence**	Triazophos	Tap water, rice, cucumber, cabbage, and apple	3.19 × 10^−11^–6.38 × 10^−8^	1.92 × 10^−11^	85.0–110	[78]
**Colorimetry**	7 pesticides simultaneously (triazophos, methyl-parathion, fenpropathrin, carbofuran, thiacloprid, chlorothalonil, and carbendazim)	Cucumber, Chinese cabbage, tomato, apple, and pear	1.21 × 10^−10^–1.46 × 10^−8^ (triazophos)	6.38 × 10^−11^ (triazophos)	73.9–116	[79]
			1.00 × 10^−8^–4.12 × 10^−7^ (methyl–parathion)	3.12 × 10^−9^ (methyl-parathion)		
			6.87 × 10^−10^–3.70 × 10^−8^ (fenpropathrin)	3.72 × 10^−10^ (fenpropathrin)		
			3.53 × 10^−8^–2.96 × 10^−7^ (carbofuran)	2.01 × 10^−8^ (carbofuran)		
			4.32 × 10^−8^–7.95 × 10^−7^ (thiacloprid)	2.55 × 10^−8^ (thiacloprid)		
			2.78 × 10^−9^–2.55 × 10^−8^ (chlorothalonil)	1.54 × 10^−9^ (chlorothalonil)		
			4.08 × 10^−10^–1.47 × 10^−8^ (carbendazim)	2.09 × 10^−10^ (carbendazim)		

**Table 3:** Electrochemical aptasensors for pesticides detection in food: main features and strategies.

Electrode	Sensing Technique and Redox Probe *	Analyte	Matrix	Linear Dynamic Range LDR (M)	Limit of Detection LOD (M)	Recovery (%)	Ref.
**MCH/aptamer/Au electrode**	EIS	Carbendazim	Soya milk, mango juice, tomato, and plum fruit	5.23 × 10^−11^–5.23 × 10^−8^	4.29 × 10^−11^	89.0–95.0	[96]
**MCH/aptamer/AuNPs/Au electrode**	EIS	Acetamiprid	Tomato	5.00 × 10^−9^–6.00 × 10^−7^	1.00 × 10^−9^	85.8–105	[97]
**MCH/aptamer/GOPTS/PtNPs microwires modified Au IDEs**	EIS	Acetamiprid and atrazine	Tap and mineral water	1.00 × 10^−11^–1.00 × 10^−7^ (acetamiprid) 1.00 × 10^−10^–1.00 × 10^−6^ (atrazine)	1.00 × 10^−12^ (acetamiprid) 1.00 × 10^−11^ (atrazine)	86.0–112 (acetam.) 79.0–113 (atrazine)	[98]
**MCH/aptamer/Ag-NG/GCE**	EIS	Acetamiprid	Cucumber and tomato	1.00 × 10^−13^–5.00 × 10^−9^	3.30 × 10^−14^	86.4–109	[99]
**MCH/aptamer (oligo 1)/AuNPs/PANI/GSPEs**	DPV (No redox probe)	Acetamiprid	Blackberry juice, peach juice, apricot juice, and apricot juice	2.50 × 10^−7^–2.00 × 10^−6^ (**NO LINEAR RANGE**)	8.60 × 10^−8^	72.5–110	[100]
**Aptamer/SA/CHIT-IO/FTO**	DPV	Malathion	Lettuce leave	3.03 × 10^−12^–3.03 × 10^−8^	3.03 × 10^−12^	80.0–88.0	[101]
**Aptamer/AMP/CuO NFs-SWCNTs/Nafion/GCE**	DPV (Methylene blue)	Chlorpyrifos	Apple and celery cabbage	2.85 × 10^−10^–4.28 × 10^−7^	2.00 × 10^−10^	96.0–107	[102]
**BSA/aptamer/Fc@MWCNTs/OMC/GCE**	CV	Chlorpyrifos	Leek lettuce and pakchoi	2.85 × 10^−9^–2.85 × 10^−4^	9.41 × 10^−10^	98.5–107	[103]
**BSA/aptamer/GO@Fe3O4/CB-CS/GCE**	CV	Chlorpyrifos	Cabbage, lettuce, leek, and pakchoi	2.85 × 10^−10^–2.85 × 10^−4^	9.41 × 10^−11^	96.0–106	[104]

* When not specified, the redox probe used is ferrocyanide/ferricyanide redox couple. MCH: 6-Mercap-1-hexanol; GOPTS: (3-glycidyloxypropyl)triethoxysilane; PtNPs: platinum nanoparticles; IDEs: interdigitated electrodes; GSPEs: graphite screen-printed electrodes; SA: streptavidin; CHIT-IO: chitosan-iron oxide nanocomposite; FTO: fluorine tin oxide; AMP: amino-modified capture probe; CuONFs: copper oxide nanoflowers; SWCNTs: single-walled carbon nanotubes; Fc: ferrocene; OMC: mesoporous carbon; GO: graphene oxide; CB: carbon black.

**Table 4:** Optical aptasensors for pesticides detection in food: main features and strategies.

Sensing Technique	Analyte	Matrix	Liner Dynamic Range LDR (M)	Limit of Detection LOD (M)	Recovery (%)	Ref.
**Colorimetry**	Phorate	Apple	1.00 × 10^−11^–1.30 × 10^−6^	1.00 × 10^−11^	93.0–105	[105]
**Colorimetry**	Malathion	Mineral water and apple	1.00 × 10^−11^–7.5 × 10^−10^	1.94 × 10^−12^		[106]
**Colorimetry**	Malathion	Apple	5.00 × 10^−13^–1.00 × 10^−9^	6.00 × 10^−14^		[107]
**Colorimetry**	Iprobenfos (IBF) and edifenphos (EDI)	unwashed and washed rice	1.00 × 10^−8^–1.00 × 10^−7^ (IBF) 5.00 × 10^−9^–2.5 × 10^−8^ (EDI)	1.00 × 10^−8^ (IBF) 5.00 × 10^−9^ (EDI)	81.1–104 (IBF) (unwashed rice) 22.3–48.4 (IBF) (washed rice) 80.5–117 (EDI) (unwashed rice) 24.3–54.8 (EDI) (washed rice)	[108]
**Colorimetry**	Malathion	Tap water and apple	1.00 × 10^−11^–7.50 × 10^−10^	5.00 × 10^−13^	89.0–110	[109]
**SERS**	Malathion	Tap water	9.99 × 10^−6^–1.01 × 10^−4^	9.99 × 10^−6^	93.9–109	[110]
**SERS**	Malathion	Tap water	5.00 × 10^−7^–1.00 × 10^−5^	5.00 × 10^−7^	87.4–111	[111]
**SERS**	Isocarbophos, omethoate, phorate and profenofos	Apple juice	0–3.80 × 10^−6^ (phorate)	3.40 × 10^−6^ (isocarbophos) 2.4 × 10^−5^ (omethoate) 4.00 × 10^−7^ (phorate) 1.40 × 10^−5^ (profenofos)		[112]
**FRET**	Edifenphos	Rice	1.61 × 10^−9^–1.93 × 10^−8^	4.19 × 10^−10^		[113]
**FRET**	Acetamiprid	Adulterated tea	5.00 × 10^−8^–1.00 × 10^−6^	3.20 × 10^−9^	97.6–102	[114]
**FRET**	Acetamiprid	Cabbage leaves	0–1.5 × 10^−7^	7.00 × 10^−10^	90.0–95.0	[115]
**Fluorescence**	Acetamiprid	Chinese cabbage	5.00 × 10^−8^–1.00 × 10^−6^	7.29 × 10^−9^	85.7–90.9	[116]

SERS: Surface-enhanced Raman Scattering; FRET: Fluorescence Resonance Energy Transfer.

**Table 5:** Electrochemical MIP sensors for pesticides detection in food: main features and strategies.

Functional Monomer	Polymerization	Electrode	Sensing Technique and Redox Probe *	Analyte	Matrix	Linear Dynamic Range LDR (M)	Limit of Detection LOD (M)	Recovery (%)	Ref.
***o*-PD**	Electrochemical	MIP/GCE	DPV (K3[Fe(CN)6])	Acephate	Tea soup	5.00 × 10^−7^–1.00 × 10^−4^	1.30 × 10^−7^	96.8–104	[138]
**Py**	Electrochemical	MIP/Au electrode	DPV (K3[Fe(CN)6])	Glyphosate	Cucumber and tapwater	2.96 × 10^−8^–4.73 × 10^−6^	1.60 × 10^−9^	72.7–99.0	[139]
**MAA**	Free radical	MIP/rGO@Au/GCE	DPV (K3[Fe(CN)6])	Carbofuran	Cabbage and cucumber	5 × 10^−8^–2.00 × 10^−5^	2 × 10^−8^	97.7–111	[140]
***p*-ATP**	Electrochemical	MIP/GR-IL-Au/CS-AuPt-NPs/GCE	DPV	Carbaryl	Apple peel and cabbage	3.00 × 10^−8^–6.00 × 10^−6^	8.00 × 10^−9^	96.0–105	[141]
***o*-PD**	Electrochemical	MIP/HPSNs-NH_2_/GCE	DPV	2,4-D	Bean sprouts	1.00 × 10^−10^–2.50 × 10^−8^	1.17 × 10^−11^	94.4–108	[142]
**APTES**	Sol-gel	MIP/Fe3O4@MWCNTs-COOH/CS/GCE	DPV	Acephate and trichlorfon	Kidney bean and cucumber	1.00 × 10^−10^–1.00 × 10^−4^ (acephate) 1.00 × 10^−11^–1.00 × 10^−5^ (trichlorfon)	6.81 × 10^−11^ (aceph.) 8.94 × 10^−12^ (trich.)	85.7–94.9	[143]
**Py**	Electrochemical	AuNP-PB-MIP/ITO	DPV (PB)	Glyphosate	Corn	2.37 × 10^−6^–7.10 × 10^−6^	5.44 × 10^−7^	97.5–101	[144]
**MAA**	Precipitation (Free radical)	MIP-IL-EGN/GCE	DPV (no redox probe)	Methyl Parathion	Cabbage and apple peel	1.00 × 10^−8^–7.00 × 10^−6^	6.00 × 10^−9^	97.0–110	[145]
**Zinc porphyrin**	MIP microspheres by free radical (precipitation polymerization)	MIPMs/AuNPs/CG/GCE	DPV (no redox probe)	Methyl parathion	Apple	8.00 × 10^−9^–1.00 × 10^−6^	3.16 × 10^−10^	96.0–100	[146]
**MB-doped *o*-phenylene-diamine**	Electrochemical	MIP/MWCNT/Pd-Ir nanocomposite/GCE	DPV (no redox probe)	Carbofuran	Cowpea, Chinese cabbage, tomato, and apple	4.00 × 10^−11^–4.00 × 10^−9^	1.70 × 10^−12^	87.5–107	[147]
**AM**	Free radical	MIP/Graphene/GCE	DPV (no redox probe)	Phoxim	Cucumber	8.00 × 10^−7^–1.40 × 10^−4^	2.00 × 10^−8^	98.1–101	[148]
**APTES**	Sol-gel	MIS (molecularly imprinted siloxane)/CPE	DPSV (no redox probe)	Dimetridazole	Egg and milk powder	1 × 10^−8^–1.00 × 10^−6^ 1.00 × 10^−6^–1.00 × 10^−4^	3.60 × 10^−9^	93.0–108	[149]
**Bromophenol blue doped *o*-aminophenol**	Electrochemical	MIP/Pt-In/GCE	DPV (no redox probe)	Imidacloprid	Tomato, cabbage, chili, and lettuce	2.00 × 10^−10^–5.00 × 10^−8^	1.20 × 10^−11^	93.6–106	[150]
***p*-ATP**	Electrochemical	MIP/AuNPs/SPCE	DPV (no redox probe)	Paraoxon	Apple and cabbage	1.00 × 10^−8^–1.00 × 10^−4^	1.00 × 10^−9^	95.2–103	[151]
**VBA**		MIP/GN/GCE	LSV (no redox probe)	Thiamethoxam	Brown rice	5.00 × 10^−7^–2.00 × 10^−5^	4.00 × 10^−8^	88.7–94.0	[152]
**VBA**		MIP/GN/GCE	LSV (no redox probe)	Imidacloprid	Brown rice	5.00 × 10^−7^–1.5 × 10^−5^	1.00 × 10^−7^	75.0–78.0	[153]
**AuNPs + *p*-ATP**	Electrochemical	FuAuNPs/ATP/MIP/AuNP-MWCNTs/GCE	LSV (no redox probe)	Methyl parathion	Tap water, apple, and cucumber	3.80 × 10^−10^–4.18 × 10^−9^ 4.18 × 10^−9^–4.18 × 10^−8^	3.04 × 10^−10^	95.2–106	[154]
***p*-ATP-FuAuNPs**	Electrochemical	MIP/MOF film/Au electrode	LSV	Glyphosate	Tap water	5.90 × 10^−15^–5.90 × 10^−9^	5.00 × 10^−15^	98.7–103	[155]
***o*-PD**	Electrochemical	MIP-rGO/GCE	LSV (no redox probe)	Imidacloprid	Pear	7.50 × 10^−7^–7.00 × 10^−5^	4.00 × 10^−7^	91.3–96.6	[156]
***p*-ABA**	Electrochemical	MIP/PB-CMK-3/GCE	LSV (PB)	Metolcarb	Cucumber, cabbage, and apple juice	5.00 × 10^−10^–1.00 × 10^−4^	9.30 × 10^−11^	92.4–98.6	[157]
**MAA**		MIP/CPE	SWV (no redox probe)	Dicloran	Tap water	1.00 × 10^−9^–1.00 × 10^−6^	4.80 × 10^−10^	94.2–96.5	[158]
**MAA**		MIP/CPE	SWV (no redox probe)	Diazinon	Tap water	5.00 × 10^−10^–1.00 × 10^−6^	4.10 × 10^−10^	94.0–96.5	[159]
**MAA**	MIP NPs by suspension polymerization	Nano-MIP/CPE	SWV (no redox probe)	Diazinon	Apple fruit	2.50 × 10^−9^–1.00 × 10^−7^ 1.00 × 10^−7^–2.00 × 10^−6^	7.90 × 10^−10^	92.5–94.7	[160]
**Itaconic acid**	Surface imprinting via controlled radical polymerization	MISP-modified SPIONs/PGE	SWSV (no redox probe)	Mancozeb	Vegetables	1.10 × 10^−8^–4.75 × 10^−7^	1.77 × 10^−9^	99.0–100	[161]
**Py**	Electrochemical	MIP/GCE	SWV (K3[Fe(CN)6])	Dimethoate	Wheat flour	1.00 × 10^−10^–1.00 × 10^−9^			[162]
**Quercetin (Qu) and Resorcinol (Re)**	Electrochemical	MIP/AuNPs/GCE	CV (no redox probe)	Methyl Parathion	Water and fruit Juice (tangerine), and vegetable juice (sweet potato leaves)	5.00 × 10^−8^–1.50 × 10^−5^	1.00 × 10^−8^	87.7–125	[163]
**MAA**	Distillation precipitation	SMIPMs/CPE	CV	Methyl Parathion	Romaine and spinach	1.00 × 10^−12^–8.00 × 10^−9^	3.40 × 10^−13^	97.2–101	[164]
**PoAP**	Electrochemical	MIP/PPy-MWCNTs-BiCo Pc/GCE	CV (K3[Fe(CN)6])	Metolcarb	Cucumber and cabbage	1.00 × 10^−8^–6.00 × 10^−7^	7.88 × 10^−9^	88.8–93.3	[165]
**MAA**		MIP/MWCNTs/Cu electrode	Potentiometry (no redox probe)	Lindane	Tap water, grape, orange, tomato, and cabbage	1.00 × 10^−9^–1.00 × 10^−3^	1.00 × 10^−10^		[166]
**MAA**		MIP/MWCNTs/IPIM	Potentiometry (no redox probe)	2,4-D	Tap water	1.00 × 10^−9^–1.00 × 10^−5^	1.20 × 10^−9^	97.6–99.2	[167]
**AM or MAA**		Sensor 1 MIP/AM/EGDMA washed Sensor 2 MIP/AM/EGDMA unwashed Sensor 3 MIP/MAA/EGDMA washed Sensor 4 MIP/MAA/EGDMA unwashed Sensor 5 Carboxylated-PVC	Potentiometry (no redox probe)	Dinotefuran	Cucumber	Sensor 1 1.00 × 10^−7^–1.00 × 10^−2^ Sensor 2 1.00 × 10^−7^–1.00 × 10^−3^ Sensor 3 1.00 × 10^−7^–1.00 × 10^−2^ Sensor 4 1.00 × 10^−7^–1.00 × 10^−3^ Sensor 5 1.00 × 10^−7^–1.00 × 10^−3^	Sensors 1 and 3 1.73 × 10^−9^ Sensor 2 4.98 × 10^−8^ Sensor 4 3.41 × 10^−8^ Sensor 5 2.13 × 10^−8^	87.9–106	[168]
**Py**	Electrochemical	MIP/PGE	EIS	Chlorpyrifos	Tap water and corn leaves	5.70 × 10^−8^–8.56 × 10^−7^	1.28 × 10^−8^	101–103	[169]
**Py**	Electrochemical	MIP/PGE	EIS (capacitance) (no redox probe)	2,4-D	Packaged drinking water and tap water	2.71 × 10^−10^–5.66 × 10^−8^	9.05 × 10^−11^	92.0–110	[170]

* When not specified, the redox probe used is ferrocyanide/ferricyanide redox couple. *o*-PD: *o*-phenylenediamine; Py: pyrrole; *p*-ATP: *p*-aminothiophenol; APTES: 3-aminopropyltriethoxysilane; MAA: methacrylic acid; MB: methylene blue; AM: acrylamide; VBA: *p*-vinylbenzoic acid; FuAuNPs: functionalized gold nanoparticles; *p*-ABA: para aminobenzoic acid; PoAP: poly-*o*-aminophenol; rGO: reduced graphene oxide; GR-IL-Au: graphene-ionic liquid-nano Au; CS-AuPtNPs: chitosan-AuPt alloy nanoparticles; HPSNs-NH_2_: hierarchical porous dendrimer-like silica nanoparticles; Fe_3_O_4_@MWCNTs-COOH/CS: Fe_3_O_4_@carboxyl-functionalized MWCNTs/chitosan nanocomposite; MIP-IL-EGN: MIP–ionic liquid–graphene composite film; MIPMs: molecularly imprinted polymer microspheres; CG: carboxylic graphene; SPCE: screen-printed carbon electrode; GN: graphene; MOFs: metal-organic frameworks; PB-CMK-3: ordered mesoporous carbon material prussian blue; CPE: carbon paste electrode; MISP: molecularly imprinted star polymers; SPIONS: superparamagnetic iron oxide nanoparticles; PGE: pencil graphite electrode; SMIPMs: surface molecularly imprinted polymeric microsphere; PPy: polypyrrole; BiCoPc: binuclear phthalocyanine cobalt(II) sulfonate; IPIM: imprinted polymer inclusion membrane; EGDMA: ethylene glycol dimethylacrylate; PVC: polyvinyl chloride.

**Table 6:** Optical MIP sensors for pesticides detection in food: main features and strategies.

Functional Monomer	Polymerization	Sensing Technique	Analyte	Matrix	Linear Dynamic Range LDR (M)	Limit of Detection LOD (M)	Recovery (%)	Ref.
**MAA**	Bulk polymerization	Fluorescence	Methamidophos	Apple and pear	3.5 × 10^−7^–7.10 × 10^−4^	9.16 × 10^−8^	89.7–94.9	[171]
**MAA**	Multi-step swelling + polymerization	Fluorescence	Carbofuran	Tap water	4.52 × 10^−9^–9.04 × 10^−8^	9.04 × 10^−10^	94.1–98.4	[172]
**MAA**		Fluorescence	Carbaryl	Rice and Chinese cabbage	4.97 × 10^−7^–3.98 × 10^−4^	1.47 × 10^−7^	74.0–88.0	[173]
**APTES**	Sol-gel polymerization	Fluorescence	2,4-D	Bean sprout (Soybean sprout and Mung bean sprout juice)	6.60 × 10^−7^–8.00 × 10^−5^	2.10 × 10^−9^	95.0–110	[174]
**APTES**	Reverse microemulsion polymerization	Fluorescence	Parathion	Tap water	5.00 × 10^−8^–1.00 × 10^−3^ (**NO LINEAR RANGE**)	2.18 × 10^−7^	99.3–100	[175]
**AM**	Precipitation polymerization	Fluorescence	Cyhalothrin	Milk	0–1.00 × 10^−4^		99.6–103	[176]
**APTES + MAA**	Modified reverse micro-emulsion	Fluorescence	Cyfluthrin	Fish	2.30 × 10^−8^–4.61 × 10^−7^	2.30 × 10^−9^	88.0–90.7	[177]
**AM**	Precipitation	Fluorescence	Cyhalothrin	Honey	0–1.00 × 10^−9^	4.00 × 10^−12^	97.0–104	[178]
**AM**	Surface imprinting fluorescent MIP spheres	Fluorescence	Cyhalothrin	Honey	0–2.50 × 10^−9^	4.00 × 10^−12^	29.0–114 (0–10nM) 94.0–114 (0–2.5nM)	[179]
**AM**	Surface imprinting technology (Free radical)	Fluorescence	Gamma-cyhalothrin	Soda water	0–5.00 × 10^−9^	5.00 × 10^−12^	96.0–111 (0–5nM) 31.0 (10nM)	[180]
**AM**	Surface molecular imprinting	Fluorescence	Gamma-cyhalothrin	Honey	0–5.00 × 10^−8^	5.11 × 10^−9^	98.0–107	[181]
**AM**	Precipitation polymerization	Fluorescence	Gamma-cyhalothrin	Tap water and Chinese spirits	0–6.00 × 10^−8^	9.17 × 10^−9^	102–106 (0–60nM) 61.4–74.2 (500nM)	[182]
**Hydroquinone doped with neutral red**	Electrochemical	Fluorescence	Fungicide fenaminosulf	Vegetables	2.00 × 10^−10^–4.00 × 10^−8^	1.60 × 10^−11^	92.0–110	[183]
**AMMB**	Precipitation polymerization (free radical)	Fluorescence	Alachlor	Corn seed	1.00 × 10^−6^–1.50 × 10^−4^	5.00 × 10^−7^	95.6–104	[184]
**7-allyloxycoumarin**	Surface molecular imprinting technique	Fluorescence	2,4,6-trichlorophenol (2,4,6-TCP)	Soda water	0–1.00 × 10^−7^	5.34 × 10^−11^	98.0–108	[185]
**MAA**	Surface molecular imprinted method (free radical)	Fluorescence	Atrazine	Tap water	2.32 × 10^−6^–1.85 × 10^−4^	8.60 × 10^−7^	77.6–115	[186]
**MR-doped *o*-phenylenediamine**	Electrochemical	FRET	Dimethoate	Chinese cabbage, broccoli and cucumber	6.00 × 10^−10^–3.40 × 10^−8^	1.83 × 10^−11^	95.0–106	[187]
**APTES + PTES**	Sol-Gel	Colorimetry	3-phenoxybenzaldehyde (3-PBD)	Fruit juice and beverage	5.04 × 10^−7^–5.04 × 10^−6^	2.62 × 10^−7^	90.0–97.8	[188]
**Zinc porphyrin and methacrylate**	Thermal polymerization	Fluorescence photometry + Colorimetry	Dimethyl methylphosphonate	Tap water	1.00 × 10^−7^–1.00 × 10^−2^ (fluorescence) 1.00 × 10^−7^–1.00 × 10^−2^ (colorimetric)	1.00 × 10^−7^ (fluor–) 1.00 × 10^−7^ (colorim.)	96.5–106	[189]
**Py**	Thermal	Electrochromism	Chlorpyrifos	Drinking water	1.00 × 10^−13^–1.00 × 10^−3^	1.00 × 10^−13^	81.0–107	[190]
**Dopamine**	Self polymerization	SPR	Chlorpyrifos	Apple	1.00 × 10^−9^–1.00 × 10^−5^	7.60 × 10^−10^	93.0–104	[191]

AMMB: 2-acrylamide-6-methoxybenzothiazole; MR: methyl red; PTES: phenyltrimethoxysilane.

## Abbreviations

2-ABA	2-aminobenzyl amine
2,4-D	2,4-dichlorophenoxyacetic acid
2,4,6-TCP	2,4,6-trichlorophenol
3-ABA	3-aminobenzoic acid
3-PBD	3-phenoxybenzaldehyde
5-DTAF	5-(4,6-dichlorotriazinyl) aminofluorescein
6-FAM-SH-ssDNAs	6-carboxyfluorescein labeled single-stranded thiol-oligonucleotides
Ab-HRP	horseradish peroxidase-labeled antibody
Ab	antibody
Ab198-cc-HRP	enzymatic labeled antibody
AET	2-aminoethanethiol
Ag	antigens
Ag	silver
AgNPs	silver nanoparticles
AM	acrylamide
AMMB	2-acrylamide-6-methoxybenzothiazole
AMP	amino-modified capture probe
ANI	aniline
Ap	aptamer
APTES	3-aminopropyltriethoxysilane
ATR-BSA	atrazine-bovine serum albumine
ATR	atrazine
Au	gold
AuNPs	gold nanoparticles
BB	bromophenol blue
BiCoPc	binuclear phthalocyanine cobalt(II) sulfonate
BSA	bovine serum albumin
CB	carbon black
CDs	carbon dots
*CdS*NPs	CdS nanoparticles
CG	carboxylic graphene
CHIT-IO	chitosan-iron oxide nanocomposite
CMK-3	ordered mesoporous carbon material
Co_3_O_4_/PANI	Co_3_O_4_/Polyaniline nanoparticles
CP	carbon paste
CPE	carbon paste electrode
CPF-BSA	Chlorpyrifos-bovine serum albumine
CPF	chlorpyrifos
CPFO	chlorpyrifos oxon
CS-AuPtNPs	chitosan-AuPt alloy nanoparticles
CS	chitosan
CuO NFs-SWCNTs	copper oxide nanoflowers and single-walled carbon nanotubes nanocomposite
CV	Cyclic Voltammetry
dc-ELISA	direct competitive enzyme-linked immunosorbent assay
DMMP	dimethyl methylphosphonate
DMZ	dimetridazole
DPV	Differential Pulse Voltammetry
EDI	edifenphos
EGDMA	ethylene glycol dimethylacrylate
EIS	Electrochemical Impedance Spectroscopy
ELISA	Enzyme Linked Immunosorbent Assay
Fc@MWCNTs-CS	ferrocene hybrid chitosan dispersed multi-walled carbon nanotubes
FDMA	ferrocenedimethylamine
Fe_3_O_4_@MWCNTs-COOH/CS	Fe_3_O_4_@carboxyl-functionalized MWCNTs/chitosan nanocomposite
Fe_3_O_4_@PDA NPs	Fe_3_O_4_@polydopamine nanoparticles
fG	graphene sheets functionalized
FITC	fluorescein 5(6)-isothiocyanate
FM	fenaminosulf
FRET	fluorescence resonance energy transfer
FTO	fluorine tin oxide
FuAuNP	functionalized gold nanoparticles
GA	glutaraldehyde
GCE	glassy carbon electrode
GECs	graphite composite electrodes
Gly	glyphosate
GN	graphene
GO	graphene oxide
GO@Fe_3_O_4_	graphene oxide@Fe_3_O_4_
GOPTS	(3-glycidyloxypropyl)triethoxysilane
GR-IL-Au	graphene-ionic liquid-nano Au
GS-MB/AuNPs	graphene sheets-methylen blue/gold nanoparticles nanocomposite
GS-PEI-Au	graphene sheets-ethyleneimine polymer-Au nanocomposites
GSPEs	graphite screen-printed electrodes
hapten-BSA	hapten-bovine serum albumin
HPSNs-NH2	hierarchical porous dendrimer-like silica nanoparticles
HRP	horseradish peroxidase
IBF	iprobenfos
IDAMs	interdigitated array microelectrodes
IDEs	interdigitated electrodes
IFE	inner filter effect
IgG-HRP	immunoglobulin G-horseradish peroxidase
IL-EGN	ionic liquid-graphene composite film
IL	ionic liquid
IMI	imidacloprid
IPIM	imprinted polymer inclusion membrane
IrOx NPs	iridium oxide nanoparticles
ITO	indium tin oxide
L-Cys	L-cysteine
LDR	linear dynamic range
LOD	limit of detection
LSPR	localized surface plasmon resonance
LSV	Linear Sweep Voltammetry
m-GEC	magnetic graphite–epoxy composite electrode
MAA	methacrylic acid
mAb	anti-triazophos monoclonal antibody
MB	methylene blue
MCH	6-Mercap-1-hexanol
MCP	micro contact printing
MCZ	mancozeb
MEPS	microextraction by packed sorbent
MH	6-mercaptohexanol
MIECS	molecularly imprinted electrochemical sensor
MIM-Zn-MAA	molecularly imprinted membrane-zinc porphyrin-mathacrylate
MIP–IL–EGN/GCE	MIP–ionic liquid–graphene composite film coated GCE
MIPMs	molecularly imprinted polymer microspheres
MIPs	molecularly imprinted polymers
MISP	molecularly imprinted star polymers
MIT	Molecular imprinting technology
MMIP	magnetic molecularly imprinted polymer
MNPs	metal nanoparticles
MOFs	metal-organic frameworks
MPS	3-(methacryloxyl) propyl trimethoxysilane
MR	methyl red
MRLs	maximum residue levels
MTMC	metolcarb
MUA	1,1-mercaptoundecanoic acid
MWCNTs	multi-walled carbon nanotubes
nano-MIP	MIP nanoparticles
NG	nitrogen doped graphene
*o*-PD	*o*-phenylenediamine
oligo2	complementary biotinylated oligonucleotide
OMC-CS	mesoporous carbon (OMC) functionalized by chitosan
OVA	ovalbumin
*p*-ABA	para aminobenzoic acid
*p*-ATP	*p*-aminothiophenol
P	pesticide solution
PAMAM	poliamidoaminic dendrimers
PANI	polyaniline
PANABA	polymer poly-(aniline-*co*-3-aminobenzoic acid)
PB-CMK-3	ordered mesoporous carbon material prussian blue
PB	prussian blue
PDA NPs	polydopamine nanoparticles
PDDA	poly (diallydimethylammonium chloride)
PGE	pencil graphite electrode
PO	paraoxon
PoAP	Poly-*o*-aminophenol
PPy	polypyrrole
PQ	paraquat
PQ1-BSAMP	Antigen biofunctionalized magnetic micro-particles
PQQ	pyrroloquinoline quinone
PTES	phenyltrimethoxysilane
PtNPs microwires	platinum nanoparticles microwires
PtNPs	platinum nanoparticles
PVC	polyvinyl chloride
Py	pyrrole
QDs	quantum dots
Qu	quercetin
Re	resorcinol
Ret	electron transfer resistance
rGO	reduced graphene oxide
rGO@Au	reduced graphene oxide and gold nanoparticles
RU	resonance unit
SA	streptavidin
SAM	self-assembled monolayer
SELEX	(selection evolution of ligands by exponential enrichment)
SERS	Surface-enhanced Raman Scattering
SiO2@FMIP	fluorescent core-shell MIP based on the surface of SiO_2_ beads
SMIPMs	surface molecularly imprinted polymeric microsphere
Sp	spermine
SPCE	screen-printed carbon electrode
SPE	screen-printed electrode
SPIONs	superparamagnetic iron oxide nanoparticles
SPR	Surface Plasmon Resonance
SWNTs	single wall nanotubes
SWSV	square wave voltammetry in stripping mode
SWV	Square Wave Voltammetry
TPD	3,5,6-trichloro-2-pyridinol
TPN-BSA	Conjugates of *N*-(pentachlorophenoxyacetyl)glycine and bovine serum albumin
UCNPs	upconversion nanoparticles
VBA	*p*-vinylbenzoic acid
